# Quantized thermoelectric Hall effect induces giant power factor in a topological semimetal

**DOI:** 10.1038/s41467-020-19850-2

**Published:** 2020-12-02

**Authors:** Fei Han, Nina Andrejevic, Thanh Nguyen, Vladyslav Kozii, Quynh T. Nguyen, Tom Hogan, Zhiwei Ding, Ricardo Pablo-Pedro, Shreya Parjan, Brian Skinner, Ahmet Alatas, Ercan Alp, Songxue Chi, Jaime Fernandez-Baca, Shengxi Huang, Liang Fu, Mingda Li

**Affiliations:** 1 Department of Nuclear Science and Engineering, Massachusetts Institute of Technology, Cambridge, MA 02139 USA; 2 Department of Materials Science and Engineering, Massachusetts Institute of Technology, Cambridge, MA 02139 USA; 3 Department of Physics, Massachusetts Institute of Technology, Cambridge, MA 02139 USA; 4 Quantum Design, Inc., San Diego, CA 92121 USA; 5 Department of Physics, Wellesley College, Wellesley, MA 02481 USA; 6 Advanced Photon Source, Argonne National Laboratory, Lemont, IL 60439 USA; 7 Neutron Scattering Division, Oak Ridge National Laboratory, Oak Ridge, TN 37831 USA; 8 Department of Electrical Engineering, The Pennsylvania State University, State College, PA 16802 USA

**Keywords:** Thermoelectrics, Topological insulators

## Abstract

Thermoelectrics are promising by directly generating electricity from waste heat. However, (sub-)room-temperature thermoelectrics have been a long-standing challenge due to vanishing electronic entropy at low temperatures. Topological materials offer a new avenue for energy harvesting applications. Recent theories predicted that topological semimetals at the quantum limit can lead to a large, non-saturating thermopower and a quantized thermoelectric Hall conductivity approaching a universal value. Here, we experimentally demonstrate the non-saturating thermopower and quantized thermoelectric Hall effect in the topological Weyl semimetal (WSM) tantalum phosphide (TaP). An ultrahigh longitudinal thermopower $S_{xx} \sim 1.1 \times 10^3 \, \mu \, {\mathrm{V}} \, {\mathrm{K}}^{ - 1}$ and giant power factor $\sim 525 \, \mu \, {\mathrm{W}} \, {\mathrm{cm}}^{ - 1} \, {\mathrm{K}}^{ - 2}$ are observed at ~40 K, which is largely attributed to the quantized thermoelectric Hall effect. Our work highlights the unique quantized thermoelectric Hall effect realized in a WSM toward low-temperature energy harvesting applications.

## Abstract (web summary)

Theories predict a large thermopower and a quantized thermoelectric Hall conductivity in topological semimetals. Here, the authors observe an ultrahigh longitudinal thermopower and a giant power factor attributed to the quantized thermoelectric Hall effect in a Weyl semimetal TaP.

## Introduction

Over two-thirds of global energy production is rejected as waste heat. Thermoelectrics are attractive by directly converting waste heat into electricity without moving parts. The efficiency of thermoelectric energy conversion is an increasing function of a dimensionless quantity $zT = \sigma S^2T/\kappa$, where *σ*, *S*, and *κ* denote the electrical conductivity, thermopower, and total thermal conductivity, respectively^1^. Conventional thermoelectrics largely focus on tuning the thermal and electrical conductivities. Many efforts, such as lowering dimensionality^2^, microstructuring^3,4^, and nanostructuring^5,6^, share the same principle: by increasing the scattering of major heat carriers of long mean free-path phonons without affecting the short mean free-path electrons, a level of independent tunability between electrical conductivity *σ* and thermal conductivity *κ* can be achieved, such as the phonon-glass electron-crystal state^7^. However, less attention was paid to improve the thermopower *S*, even though the *S*^*2*^ dependence in *zT* makes such improvement appealing. Moreover, thermopower *S* is proportional to the entropy per carrier and is therefore suppressed at reduced temperature^8^. For this reason, current thermoelectrics are generally effective only at elevated temperatures and there is a pressing need for thermoelectrics that work efficiently at room temperature and below. Filling this need requires new materials that can exhibit large electronic entropy at low temperatures while maintaining significant electrical conductivity.

One approach to creating large electronic entropy is bandstructure engineering through low carrier density, partially filled carrier pockets^9^; a similar principle has also been applied to semimetals, such as Bi^10^, graphite^11^, and most recently Weyl semimetals (WSMs), to explore large entropy at low carrier density^12–14^. However, the electrical conductivity is thereby reduced. Magnetic field offers an additional incentive to dramatically increase the entropy, as the linear field dependence of the density of states (DOS) enables unbounded, macroscopic number of states in each Landau level (LL), yet in conventional thermoelectrics, charge carriers will be localized at high *B*-field due to the cyclotron motion, still resulting in low conductivity. Consequently, increasing power factor $\left( { \equiv \sigma S^2} \right)$ creates a significant challenge as it requires optimization of both *σ* and *S* under conflicting conditions.

The recent development of topological materials^15,16^, including topological WSMs^17^, offers a new pathway to surpass conventional thermoelectrics that relies on the topological protection of electronic states^18,19^. It is particularly worthy to note that the WSM system has a unique *n* = 0 LL, which has a highly unusual, energy-independent DOS $g(n = 0) = N_fBe/4\pi ^2\hbar ^2v_F$ increasing linearly with *B*, and therefore can create huge electronic entropy. More importantly, the system remains gapless under high field, thanks to the topological nature of Weyl nodes. Consequently, recent theories predicted a non-saturating thermopower^20^ and quantized thermoelectric Hall conductivity at the quantum limit^21^, where electrons and holes contribute additively to high thermoelectric performance without experiencing localization.

**Fig. 1:**
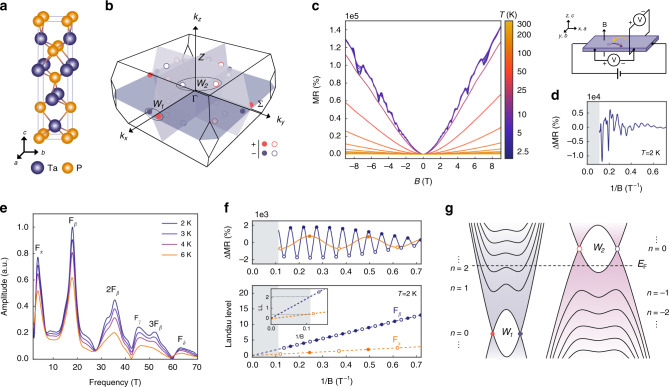
Quantum oscillation of TaP. **a** The inversion-symmetry-breaking crystal structure and **b** the Brillouin zone of TaP, highlighting the locations of the inequivalent Weyl nodes W1 (filled circles) and W2 (empty circles). The Weyl nodes are paired as source “+” (red) and sink “−” (blue) of Berry curvature, separated in momentum space. **c** Magnetoresistance (MR) as a function of magnetic field at different temperatures from 2.5 K to 300 K. A high (>10^5^%) MR ratio is observed. **d** The MR measurement configuration (top) and Δ*MR* as a function of 1/B (bottom). e^−^ and h^+^ denote electrons and holes, respectively. **e** The Fourier transform of Δ*MR* showing a low oscillation frequency $F_\alpha = 4{\mathrm{T}}$. This is a signature that, in addition to the electron pocket from W1 Weyl node contributing to $F_\beta = 18{\mathrm{T}}$, we are very close to the W2 Weyl node. **f** The SdH oscillation and Landau level index plot, from which we obtained an *n* = 2 Landau level and another *n* = 0 Landau level. **g** The schematic bandstructure at finite magnetic fields of our TaP sample.

In this work, we carry out high-precision thermoelectric measurements using a centimeter-sized crystal WSM TaP (Fig. 1a, b and Supplementary Notes 1 and 2). The Fermi level is fine-tuned through the synthesis procedure to approach the *n* = 0 LL near the W2 Weyl node (Fig. 1g). In this system, giant, non-saturating longitudinal thermopower *S*_*xx*_ is observed, which exhibits linear dependence with *B*-field without saturation. In addition, the signature of the quantized thermoelectric Hall conductivity is observed, where the low-temperature, high-field thermoelectric Hall conductivity $\alpha _{xy} \equiv \left[ {\rho ^{ - 1}S} \right]_{xy}$ approaches a universal curve determined by number of fermion flavors, Fermi velocity, and universal constants. Moreover, evidence of Wiedemann–Franz (WF) law violation further indicates a breakdown of quasiparticle behaviors. Our work leverages the effects of topology to overcome challenges for low-temperature thermoelectric energy harvesting from a power factor perspective.

## Results

### Quantum oscillations

We first present the longitudinal magnetoresistance (MR) data, where Giant MR was observed. At *T* < 25 K, the $MR \equiv \left( {\rho _{xx}(B) - \rho _{xx}(0{\mathrm{T}})} \right)/\rho _{xx}(0{\mathrm{T}})$ exceeds 10^5^% (Fig. 1c). This is a signature of electron-hole compensation, which is further confirmed by the two-band model fitting of conductivity, with $n_e = 2.39 \times 10^{19} \, {\mathrm{cm}}^{ - 3}$ and $n_h = 2.35 \times 10^{19} \, {\mathrm{cm}}^{ - 3}$ at *T* = 2.5 K, along with a high mobility of $\sim 1 \times 10^5 \, {\mathrm{cm}}^2 \, {\mathrm{V}}^{ - 1} \, {\mathrm{s}}^{ - 1}$(Supplementary Note 3). The background-subtracted MR, denoted Δ*MR*, exhibits Shubnikov-de Haas oscillations, which are plotted against 1/*B* to determine oscillation frequencies (Fig. 1d). The Fourier transform of Δ*MR* shows two small carrier pockets with low frequency $F_\alpha = 4{\mathrm{T}}$ and $F_\beta = 18{\mathrm{T}}$ among four pockets (Supplementary Note 4 and Fig. 1e). The LL fan diagram analysis indicates the two small pockets are at *n* = 2 LL and *n* = 0 LL, respectively (Supplementary Note 5 and Fig. 1f). The intersections of the linear LL index plots (−0.037 for *n* = 0 LL and +0.065 for *n* = 2) lying between −1/8 to +1/8 indicates that the two pockets are both topologically nontrivial^22,23^, from which we attribute the *n* = 2 LL to the electron pocket of the W1 Weyl node, and the *n* = 0 LL to the hole pocket of the W2 Weyl node (Fig. 1g). Moreover, we see that the W2 and W1 pockets enter the quantum limit at *B* ~ 3.8 T and 16 T, respectively. There is an alternate way to infer LL. The Weyl fermion dispersion of the *n*^th^ LL at is given by $E_{n} = {\mathrm{sgn}}(n)v_{F}\sqrt {2e\hbar B\left| n \right|}$, whereas the oscillation frequency *F* satisfies $F = E_{F}^2/2e\hbar v_{{F}}^2$. When $E_{n}\sim E_{F}$, we have $F\sim B\left| n \right|$. This leads to an agreement between *n* = 2 LL and the measured $F_\beta = 18{\mathrm{T}}$ at *B* ~ 9 T. For $F_\alpha$, the low frequency 4 T suggests an extremely small Fermi surface. Since the spacing between *n* = 1 and *n* = 0 LLs is given by $E_1 - E_0 = v_{F}\sqrt {2e\hbar B} = E_{F}\sqrt {B/F}$, the condition to reach the *n* = 0 LL quantum limit for W2 pocket is met at $B\, > \, F_\alpha = 4{\mathrm{T}}$. This value agrees well with the above LL index analysis.

### Non-saturating thermopower and giant power factor

**Fig. 2:**
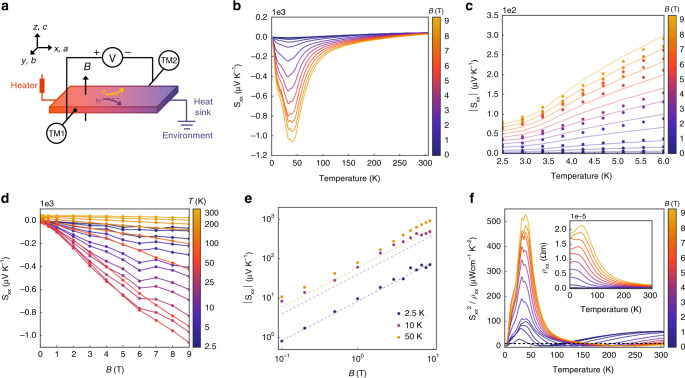
Non-saturating thermopower at high fields. **a** The schematics of the diagonal offset thermoelectric measurement geometry. TM1 and TM2 represent thermometer 1 and 2. The temperature difference between the short ends of the sample is represented by the color gradient from red (high) to blue (low). **b** Longitudinal thermopower *S*_*xx*_ as a function of temperature at various fields. The double peaks emerge at ~33 K and ~40 K. **c** *S*_*xx*_ in the low-temperature range, showing the quasi-linear growth as a function of temperature. **d** *S*_*xx*_ replotted as a function of *B*, showing unbounded linear growth with field. The onset of the linear behavior indicates entrance into the quantum limit regime. The oscillatory behavior ~20 K at *B* = 6 T is caused by the quantum oscillation effect. **e** *S*_*xx*_ as a function of *B* at a few representative temperatures. The dashed lines are theoretical values using Eq. (1) by substituting the fitted *v*_*F*_ from Eq. (3) (for *T* = 2.5 K) and Eq. (S14) (for *T* ≥ 10 K). **f** The power factor as a function of temperature. The black-dashed line is a reference peak value for SnSe.

Having determined the carrier characteristics, we carried out thermoelectric measurements using a diagonal offset geometry (Fig. 2a), where the electrical and thermal transport along both the longitudinal and transverse directions can be acquired together by flipping the field polarity (Supplementary Note 6, which also contains the phase relations between various thermoelectric quantities). The longitudinal thermopower *S*_*xx*_ is shown in Fig. 2b, where *S*_*xx*_ increases over 200-fold compared to its zero-field value, and reaches a giant magnitude of $1.07 \times 10^3 \, \mu {\mathrm{VK}}^{{\mathrm{ - 1}}}$ without sign of saturation at *B* = 9 T and *T* = 40 K. One prominent feature is that *S*_*xx*_ develops a double-peak behavior, which may be attributed to the two types of Weyl nodes: the higher carrier mobility and lower carrier density at the W2 node leads to reduced phonon scattering, and thus the high *S*_*xx*_ can persist to higher temperatures. Quantitatively, it has been predicted that for the *n* = 0 chiral LL of Weyl electrons, *S*_*xx*_ obeys a simple formula^20^:

1 $$S_{xx} = \frac{k_{B}^2}{{h^2}}\frac{{N_{f}}}{{12}}\frac{{{TB}}}{{v_{F}^{{\mathrm{eff}}}\left( {n_{h} - n_{e}} \right)}}$$

where *N*_f_ is the degeneracy of the Weyl nodes, $n_{h} - n_{e}$ is the net carrier density, and $v_{F}^{{\mathrm{eff}}}$ is an effective Fermi velocity. As TaP has two sets of Weyl nodes with different velocities and energies, in this work we introduce $v_{F}^{{\mathrm{eff}}}$ as an effective parameter capturing an average Fermi velocity of the system.

The linearity of *S*_*xx*_ with *T* and *B* is shown in Fig. 2c, d, respectively. It is noteworthy that Eq. (1) is in quantitative agreement with our result if we adopt the fitted value of the $v_{F}^{{\mathrm{eff}}}$ using Eq. (3) and Eq. (S14), described in greater detail in the following section. Such quantitative agreement is valid across all fields and up to ~40 K and is a measure of the success of the effective model (Fig. 2e). Moreover, a giant longitudinal power factor $\equiv S_{xx}^2/\rho _{xx}$ up to 525 *μ*W cm^−1^ K^−2^ is achieved due to the large entropy generated by the linearly-dispersive bands at quantizing magnetic fields, while a low *ρ*_*xx*_ is maintained due to the protection of the gapless *n* = 0 LL, evading the typical fate of carrier cyclotron motion and localization under fields^20,21^. In fact, this value is an order of magnitude higher than peak values of promising thermoelectrics (e.g., 10 *μ*W cm^−1^ K^−2^ for SnSe at ~800 K^24^) and two orders of magnitude higher than non-topological semimetals^10,11^, which can achieve high thermopower at high magnetic fields with linear-dispersive bands, but cannot simultaneously maintain a low magneto-electrical resistivity.

### Quantized thermoelectric Hall effect

**Fig. 3:**
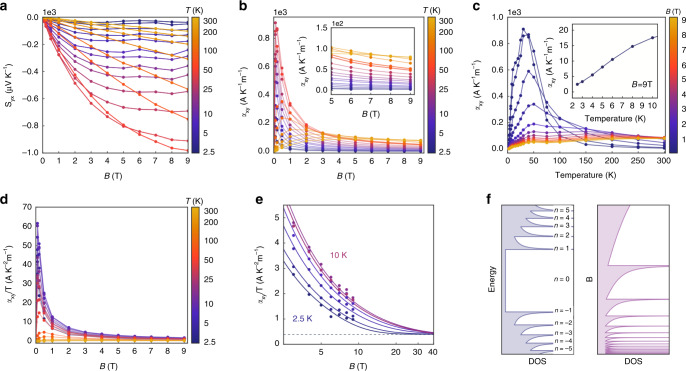
The quantized thermoelectric Hall effect. **a** Transverse thermopower *S*_*yx*_ as a function of magnetic field at different temperatures. **b** Thermoelectric Hall conductivity $\alpha _{xy}$ as a function of magnetic field at different temperatures. The peak value is caused by the finite scattering effect. **c** Thermoelectric Hall conductivity $\alpha _{xy}$ as a function of temperature at various fields. The inset shows a linear behavior of $\alpha _{xy}$ versus *T* at low temperatures. **d** $\alpha _{xy}/T$ as a function of magnetic field at various temperatur**e**s. **e** An extrapolation of **d** showing a convergence to the quantized value at low temperatures. **f** The density of states (DOS) of each Landau level (LL), highlighting the unique *n* = 0 LL in a WSM. At high-enough *B*, *n* = 0 LL drives the DOS ∝ *B*.

Regarding the transverse properties, we see that the transverse thermopower *S*_*yx*_ exhibits a plateau with increasing *B-*field, which is known to originate from the constant *k*-space volume as thermopower is a measure of occupational entropy in state space^12^. The thermoelectric Hall conductivity $\alpha _{xy} \equiv ( {S_{xx}\rho _{yx} - S_{yx}\rho _{xx}} )/( {\rho _{xx}^2 + \rho _{yx}^2} )$ is shown in Fig. 3b, where in the low-temperature range, the flatness with respect to *B-*field starts to emerge. In particular, under the low-temperature $k_{B}T \ll E_{F}$ and high-field $B \gg E_{F}^2/\hbar ev_{F}^2$ limit, *α*_*xy*_ is predicted to approach the following universal value that is independent of *B*-field, disorder, carrier density, and even carrier type^21^:

2 $$\alpha _{xy,\;{\mathrm{ideal}}} = \frac{{\pi ^2}}{3}\frac{{ek_{B}^2}}{{(2\pi \hbar )^2}}\frac{{N_{f}}}{{v_{F}^{{\mathrm{eff}}}}}T$$

**Fig. 4:**
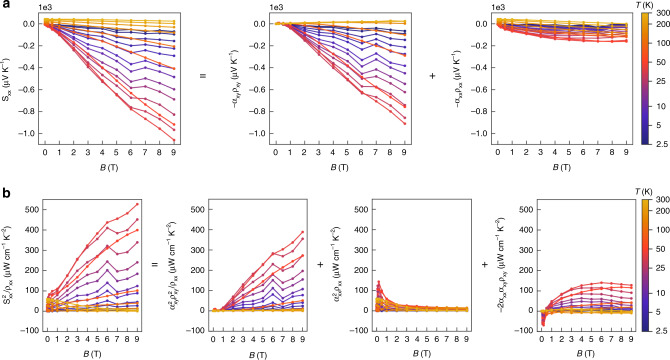
Dominant contribution from the transverse thermoelectric Hall conductivity to longitudinal thermopower and power factor. **a** Total $S_{xx}$ (left-hand side, LHS) at various temperatures as a function of magnetic field and its transverse component $- \alpha _{xy}\rho _{xy}$ (first term on the right-hand side, RHS) and longitudinal contribution $+ \alpha _{xx}\rho _{xx}$ (second term on the RHS). **b** Total $S_{xx}^2/\rho _{xx}$ (LHS) at various temperatures as a function of magnetic field and its transverse component $+ \alpha _{xy}^2\rho _{xy}^2/\rho _{xx}$ (first term on RHS), longitudinal contribution $+ \alpha _{xx}^2\rho _{xx}$ (second term on the RHS), and the cross-term $- 2\alpha _{xy}\alpha _{xx}\rho _{xy}$ (third term on the RHS). The dominant contribution of transverse component can be seen at all temperatures.

The temperature dependence of *α*_*xy*_ is shown in Fig. 3c, where we see that the linearity with *T* holds up to *T* ~ 10 K. As a direct consequence, the $\alpha _{xy}/T$ curves converge to a single curve at high fields (Fig. 3d, e), where an ideal value $\alpha _{xy,\;{\mathrm{ideal}}}/T = 0.4 \, {\mathrm{A}} \, {\mathrm{K}}^{ - 2} \, {\mathrm{m}}^{ - 1}$ is determined by evaluating Eq. (2) using $v_{F}^{{\mathrm{eff}}} = 1.4 \times 10^4 \, {\mathrm{m}} \, {\mathrm{s}}^{ - 1}$, which is extracted by fitting a more general Eq. (3) at base temperature:

3 $$\alpha _{xy} = \frac{{eN_{f}}}{{2\pi \hbar }}\mathop {\sum}\limits_{n = 0}^\infty \int_0^\infty {\frac{{dk_z}}{\pi }\left[ {s\left( {\frac{{\varepsilon _n^0(k_z) - \mu }}{{k_{B}T}}} \right) + s\left( {\frac{{\varepsilon _n^0(k_z) + \mu }}{{k_{B}T}}} \right)} \right]}$$

in which *s* is the electronic entropy function (Eq. S13). The magnitude $v_{F}^{{\mathrm{eff}}}$ is comparable to the simple weighted average of projected Fermi velocity $v_{F,z}^{W1} = 0.77 \times 10^5 \, {\mathrm{m}} \, {\mathrm{s}}^{ - 1}$, $v_{F,z}^{W2} = 1.88 \times 10^5 \, {\mathrm{m}} \, {\mathrm{s}}^{ - 1}$^25^, which gives $\bar v_{F,z}^{{\mathrm{eff}}} = 1.5 \times 10^5 \, {\mathrm{m}}{\,}{\mathrm{s}}^{ - 1}$, where the *z*-direction was chosen to coincide with the magnetic field direction. The fitted chemical potential *μ* is consistent with the electrical transport measurements, whereas the $v_{F}^{{\mathrm{eff}}}$ is lower than the *v*_*F*_ at W2^25^. This can be understood, as carriers at W1 Weyl nodes at *n* = 2 LL have yet to reach extreme quantum limit (Figs. 1g and 3f, and Supplementary Notes 6 and 7). For temperatures above 10 K, scattering effects are significant and the dissipationless limit assumed in Eq. (3) is no longer valid; thus, for fits at *T* ≥ 10 K, approximate forms of $\alpha _{xy}$, which include a finite scattering time were used (Eq. (S14) and Eq. (S16)). To corroborate the universal quantization behavior of $\alpha _{xy}/T$, we performed separate thermoelectric measurements up to *B* = 14 T at *T* = 2 K, 4 K and 6 K, where the collapse onto a single curve and a clearer plateau are observed (Supplementary Note 8), in addition to giving $\alpha _{xy,\;{\mathrm{ideal}}}/T = 0.37 \, {\mathrm{A}} \, {\mathrm{K}}^{ - 2} \, {\mathrm{{m}}}^{ - 1}$, in quantitative agreement with the 9 T data. Finally, to show that the quantized thermoelectric Hall coefficient $\alpha _{xy}$ drives the ultrahigh thermopower and giant power factor, we decompose $S_{xx}$ into its transverse $( { - \alpha _{xy}\rho _{xy}})$ and longitudinal $\left( {\alpha _{xx}\rho _{xx}} \right)$ components, where we see that the transverse term $\alpha _{xy}\rho _{xy}$ contributes to ~90% of the longitudinal $S_{xx}$ (Fig. 4a and Supplementary Note 9). The corresponding decomposed contributions to power factor $S_{xx}^2/\rho _{xx}$ is shown in Fig. 4b.

In a nutshell, the quantized $\alpha _{xy}$, large non-saturating $S_{xx}$, and ultrahigh power factor $S_{xx}^2/\rho _{xx}$ all originate from the topological Weyl nodes, but with increasingly stringent manifestation conditions: the quantized $\alpha _{xy}$ comes directly from the gapless *n* = 0 LL states of Weyl fermions; as $S_{xx} = - \alpha _{xy}\rho _{xy} + \alpha _{xx}\rho _{xx}$, $\rho _{xy}$ should increase at high fields to obtain non-saturating $S_{xx}$ with the field-independent $\alpha _{xy}$; only when the transverse components $- \alpha _{xy}\rho _{xy}$ dominate the $S_{xx}$ with moderate $\rho _{xx}$ can the power factor $S_{xx}^2/\rho _{xx}$ be enhanced; the gapless *n* = 0 LL states can also contribute to reduce the $\rho _{xx}$.

### Breakdown of the Wiedemann-Franz law

The Wiedemann-Franz law is a robust empirical law stating that the ratio between the electronic thermal conductivity $\kappa ^e$ and electrical conductivity *σ* are related by a universal Lorenz number:

4 $$L_0 \equiv \frac{\kappa ^{e}}{\sigma T} = \frac{\pi ^{2}}{3}\left( \frac{{k_{B}}}{e} \right)^2 = 2.44 \times 10^{ - 8}{\,}{\mathrm{{W}}} \, \Omega \, {\mathrm{K}}^{ - {\mathrm{2}}}$$

**Fig. 5:**
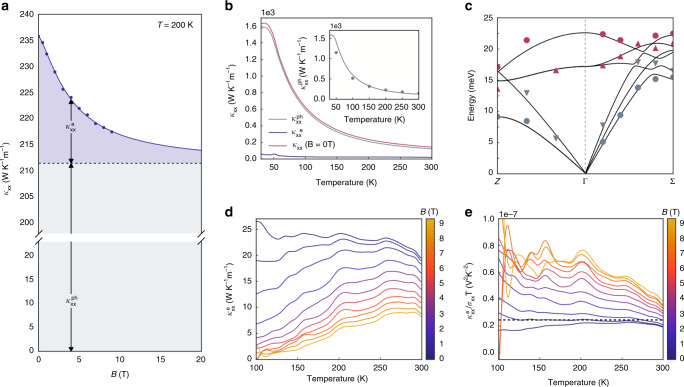
The Wiedemann–Franz Law. **a** The schematics of the separation process of electronic thermal conductivity $\kappa _{xx}^e$ from the lattice thermal conductivity using $\kappa _{xx}^{ph}$ the field dependence. **b** Separation of phonon and electronic contributions to the longitudinal thermal conductivity with inset displaying a computation (scattered points) of the phonon thermal conductivity from first principles. **c** Experimentally measured values of phonon modes (scattered points) of TaP along high-symmetry line Z-Γ-Σ taken by inelastic *x*-ray and neutron scattering with accompanying ab initio calculation (solid lines), displaying good agreement between ab initio calculations and experiment. **d** The electronic contribution of the thermal conductivity as a function of temperature at various fields. **e** The Lorenz number as a function of temperature at various fields. The black line indicates the theoretical value of the Wiedemann–Franz law.

Recently, it has been reported that there is strong violation of the WF law in the 2D Dirac fluid of graphene^26^ and WSM WP_2_^27^ due to collective electron hydrodynamics. Other behaviors of electrons, like quantum criticality^28^ or quasiparticle breakdown^29,30^, can also lead to the WF law violation. It is thus worthwhile to examine the validity of the WF law in the field-induced high-entropy state of TaP. To do so, it is crucial to properly separate $\kappa ^e$ from the lattice thermal conductivity $\kappa ^{ph}$. We adopt the following empirical relation by using the field-dependence of $\kappa ^e$^31^:

5 $$\kappa _{xx}\left( {T,\;B} \right) = \kappa _{xx}^{ph}\left( T \right) + \frac{{\kappa _{xx}^e\left( T \right)}}{{1 + \beta _e\left( T \right)B^m}}$$

where $\beta _e(T)$ is a measure of zero-field electron mean free path and *m* is a factor related to the nature of scattering. Figure 5a demonstrates an example for such a separation procedure (Supplementary Note 11). Using this method, we see that the extracted $\kappa ^{ph}$ agrees well with the computed value from ab initio calculations (Fig. 5b and Supplementary Note 12), from which the phonon dispersions are also computed, and agree well with measured dispersion from inelastic scattering (Fig. 5c and Supplementary Note 10). All these agreements indicate the reliability of the separation process. The corresponding $\kappa ^e$ and the $L_0$ is shown in Fig. 5d, e, respectively. At *B* = 0 T, the agreement with the WF law is good. However, as field increases to *B* = 9 T, a fourfold violation of WF law is observed (Fig. 5d). This happens across a wide temperature range but not at low temperatures, indicating the link of scattering (Supplementary Note 11). The observed strong violation of the WF law hints at the possibility of field-driven, scattering-enhanced collective behaviors in a large entropic system and is subject to further investigation.

## Discussion

### Pathway toward room-temperature topological thermoelectrics

In this work, we report high thermopower and giant thermoelectric power factor in the WSM TaP, induced by the quantized thermoelectric Hall effect originating from topologically protected Weyl nodes at the quantum limit. These features are linked as follows: in a strong magnetic field, $S_{xx}\sim \alpha _{xy}\rho _{yx}$, the quantizing behavior of *α*_*xy*_ combined with the continual increase of *ρ*_*yx*_ with magnetic field leads to the growth of *S*_*xx*_, while the suppression of longitudinal portion $\alpha _{xx}\rho _{xx}$ to $S_{xx}$ further contributes to high power factor $S_{xx}^2/\rho _{xx}$. The choice of TaP is due to its simpler Fermi surface compared to other members in the TaAs family^25^. On the other hand, the huge mass difference between Ta and P atoms reduces the three-phonon process and results in a high thermal conductivity, making it not directly applicable as a thermoelectric material. Even so, our work sheds light on a systematic pathway to seek promising topological thermoelectrics: To increase $S_{xx}$, large carrier compensation is desired (Eq. (1)). To simultaneously maintain low $\rho _{xx}$, simultaneously high carrier densities $n_h$ and $n_e$ are required but not sufficient. In a topologically trivial semimetal such as Bi, although high thermopower can be achieved at the quantum limit $\left( {S_{xx}({\mathrm{Bi}})\sim 3 \times 10^3 \, \mu {\mathrm{V}} \, {\mathrm{K}}^{ - 1}\;{\mathrm{vs}}\;S_{xx}({\mathrm{TaP}})\sim 1 \times 10^3 \, \mu {\mathrm{V}} \, {\mathrm{K}}^{ - 1}} \right)$, the electrical resistivity is significantly enhanced at high magnetic field $\left( {\rho _{xx}({\mathrm{Bi}})\sim 2 \times 10^{ - 2} \, {\Omega}{\mathrm{m}}\;{\mathrm{vs}}\;\rho _{xx}({\mathrm{TaP}})\sim 1 \times 10^{ - 5} \, {\Omega}{\mathrm{m}}} \right)$^10^, indicating the crucial contribution of the gapless *n* = 0 LL states from the topologically protected Weyl nodes. To tune the working temperature toward room temperature, long relaxation time is favored, along with preservation of the quantum limit, where thermal energy is smaller than the LL level spacing, $k_{B}T \ll v_{F}\sqrt {\hbar eB}$^21^. Finally, intrinsic magnetism can be used to replace the external *B-*field. Overall, we foresee that magnetic topological WSMs and related topological nodal line semimetals^32,33^ with protected gapless states are promising candidate materials for thermoelectrics when charge carriers are largely compensated and the Fermi level is tuned to the gapless nodes to unlock the quantized thermoelectric Hall effect. To summarize, we demonstrated non-saturating longitudinal thermopower, giant power factor, and a signature of quantized thermoelectric Hall conductivity in a WSM in quantitative agreement with recent theoretical proposals. Furthermore, a field-driven breakdown of the WF law is observed at intermediate temperatures. Given the promising magnitudes of thermopower and power factor, our work sheds light on a few essential requirements that high-performance room-temperature thermoelectrics should meet. These include a way to create giant electronic entropy and reduce carrier density, and a way of evading localization while maintaining high electrical conductivity. Interestingly, the *n* = 0 LL state with topologically protected Weyl nodes in a WSM satisfies all these requirements. Our work thus demonstrates the possibility of topological materials to lead the breakthrough of thermoelectric materials working below room temperature.

### Note added

When we were finalizing this manuscript, we became aware of a work on Dirac semimetal^34^. The related work and our work mutually strengthened each other on the part of the quantized thermoelectric Hall effect.

## Supplementary information

Supplementary Information

## Data Availability

The datasets generated during and/or analysed during the current study are available from the corresponding author on reasonable request.
