# Supplementary material for: Quantized thermoelectric Hall effect induces giant power factor in a topological semimetal
Source: Nat Commun. 2020 Dec 2;11:6167. doi: 10.1038/s41467-020-19850-2 (PMC7710760; doi:10.1038/s41467-020-19850-2)
Supplement: Supplementary file 1 — Supplementary Information [file 41467_2020_19850_MOESM1_ESM.pdf]

Quantized Thermoelectric Hall Effect Induces Giant Power Factor in a Topological Semimetal: Supplementary Information

Fei Han[1*†], Nina Andrejevic[2†], Thanh Nguyen[1†], Vladyslav Kozii[3†], Quynh T. Nguyen[1,3], Tom Hogan[4], Zhiwei Ding[2], Ricardo Pablo-Pedro[1], Shreya Parjan[5], Brian Skinner[3], Ahmet Alatas[6], Ercan Alp[6], Songxue Chi[7], Jaime Fernandez-Baca[7], Shengxi Huang[8], Liang Fu[3*], Mingda Li[1*]

[1]Department of Nuclear Science and Engineering, Massachusetts Institute of Technology, Cambridge, MA 02139, USA

[2]Department of Materials Science and Engineering, Massachusetts Institute of Technology, Cambridge, MA 02139, USA

[3]Department of Physics, Massachusetts Institute of Technology, Cambridge, MA 02139, USA

[4]Quantum Design Inc, San Diego, CA 92121

[5]Department of Physics, Wellesley College, 106 Central St, Wellesley, MA 02481, USA

[6]Advanced Photon Source, Argonne National Laboratory, Lemont, IL 60439, USA

[7]Neutron Scattering Division, Oak Ridge National Laboratory, Oak Ridge, TN, 37831, USA

[8]Department of Electrical Engineering, The Pennsylvania State University, State College, PA 16802, USA

[†]These authors contribute equally to this work.

*Corresponding authors: hanfei@mit.edu; liangfu@mit.edu; mingda@mit.edu.

Contents

1. High-quality Single-crystal Growth

2. Sample Preparation for Measurements

3. Carrier Concentration and Mobility

4. Analysis of Quantum Oscillation

5. Landau Level and Quantum Limit

6. Data Analysis for Thermoelectric Measurement

7. Thermoelectric Hall Conductivity up to 9T

8. Low-temperature Thermoelectric Measurements up to 14T

9. Dominant Thermoelectric Hall Contribution to Longitudinal Thermoelectric Performance at Low Temperatures

10. X-Ray and Neutron Scattering Measurement Details

11. Separation of Phonon and Electron Contributions to Thermal Conductivity

12. Computational Details

1. High-quality Single-crystal Growth

We successfully obtained centimeter-sized single crystals of TaP using the vapor transport method. The single crystals of TaP were prepared in two steps. In the first step, 3 grams of Ta (Beantown Chemical, 99.95%) and P (Beantown Chemical, 99.999%) powders were weighed, mixed, and ground in a glovebox. The mixed powders were flame-sealed in a quartz tube which was subsequently heated to 700°C and dwelled for 20 hours for a pre-reaction. In the second step, the obtained TaP powders were sealed in another quartz tube with 0.4 grams of I_2 (Sigma Aldrich, >=99.8%) added. The tube containing TaP and I_2 was then horizontally placed in a two-zone furnace. To improve the crystal size and quality, instead of setting a 100°C temperature difference, we

gradually increased the temperature difference from zero until the I_2 transport agent started to flow. This process seems to be furnace- and distance-specific. In our case, the optimal temperatures for the two zones are 900°C and 950°C, respectively, and the distance between the two heating zones is constantly optimized. With the help of the transport agent I_2, the TaP source materials transferred from the cold end of the tube to the hot end and condensed at the hot end in a single-crystalline form in 14 days. The resulting products of TaP single crystals are centimeter-sized and have a metallic luster. A typical crystal is shown in Figure S1.

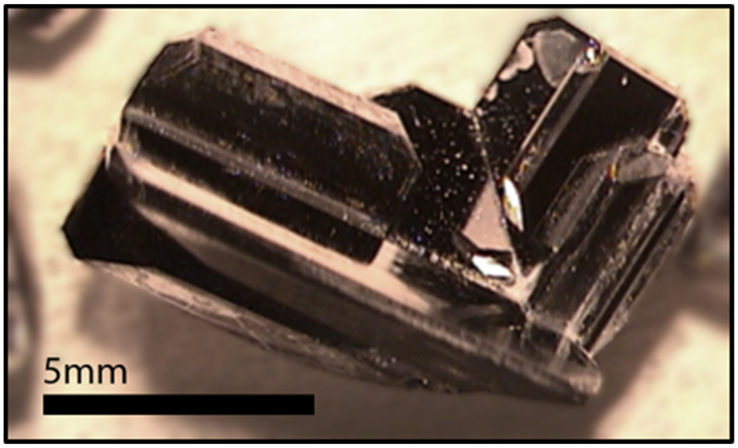

Figure S1. **Single crystals of TaP grown by the vapor transport method.**

2. Sample Preparation for Measurements

To conduct high-precision electrical and thermal transport measurements on TaP, we performed a thinning-down process on the crystals. Due to the very high electrical and thermal conductivities of TaP, it is difficult to do high-precision electrical and thermal transport measurements on the as-grown crystals. To magnify the electrical resistance and the temperature gradient in the electrical and thermal transport measurements, one piece of crystal was polished down to a thin slab along the *c*-axis. Figures S2a and b display top and side views of the thinned-down crystal we used for the thermal transport

measurement (namely thermoelectric measurement) whose thickness is only 0.17 mm. Figure S2c shows the probe configuration on the thinned-down crystal for the thermoelectric measurement, and Figures S2d and e give explanatory schematics to the usages of the probes in the thermal conductivity, thermopower, and resistivity acquisitions. The contacts of the probes were made with the silver epoxy H20E.

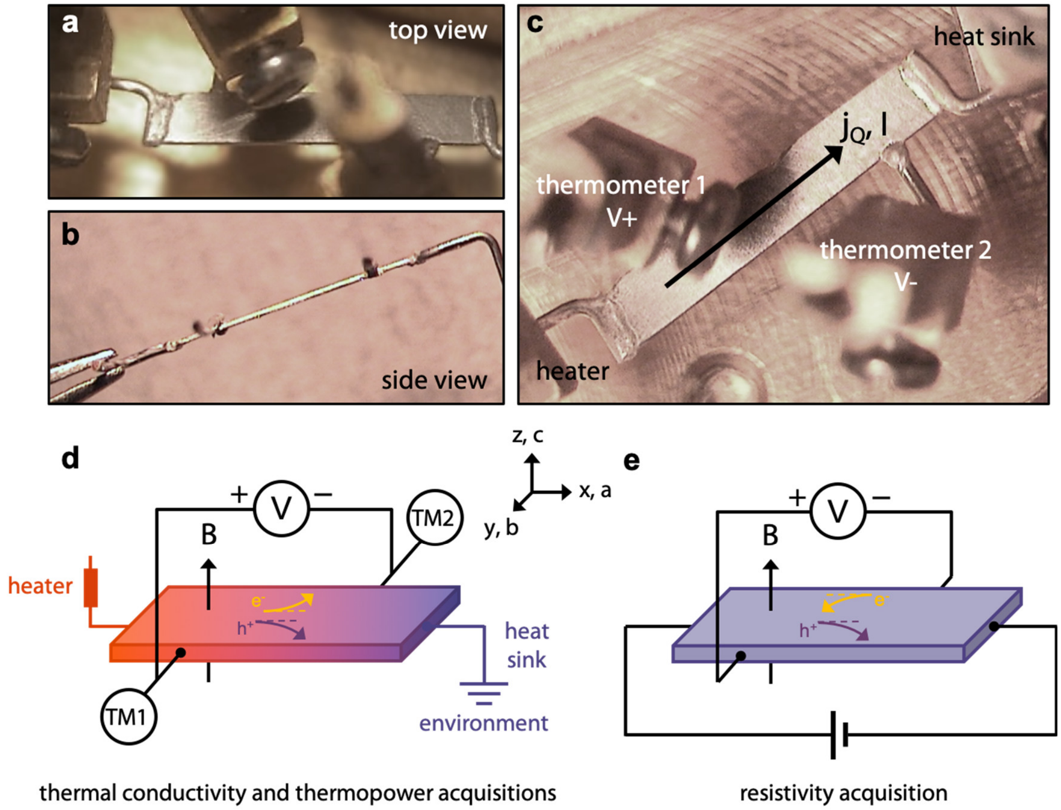

Figure S2. **Thermoelectric measurement configuration.** **a** Top and **b** side view of the thinned-down TaP crystal we used for the thermoelectric measurement. The thickness is as thin as 0.17mm. **c** Probe configuration on the thinned-down crystal for the thermoelectric measurements. Explanatory schematic diagrams for the usage of the probes **d** in the thermal conductivity and thermopower acquisitions and **e** in the resistivity acquisition. TM1 and TM2 represent thermometer 1 and 2. The temperature difference between the short ends of the sample in **d** is represented by the color gradient from red (high) to blue (low). e- and h+ denote electrons and holes, respectively.

3. Carrier Concentration and Mobility

The electrical and thermal transport measurements were carried out with the electrical transport option (ETO) and the thermal transport option (TTO) of physical property measurement system (PPMS), respectively. The data about the quantum oscillations were measured with the ETO whereas the data about the thermoelectric (including resistivity) with the TTO. When we performed the ETO measurements we adopted a standard six-probe configuration and connected the longitudinal and transverse probes to two independent measurement channels. The details about the ETO measurement can be found in Figure S3a. However, because the TTO has only one measurement channel, to measure the longitudinal and transverse thermal conductivities (κ_{xx} and κ_{yx}), resistivities (ρ_{xx} and ρ_{yx}), and Seebeck coefficients (S_{xx} and S_{yx}) simultaneously, we used a diagonal offset probe geometry for the thermal transport measurement, as shown in Figures S7a and S8a. For the detailed description about the TTO measurement, consult Supplementary Information VI.

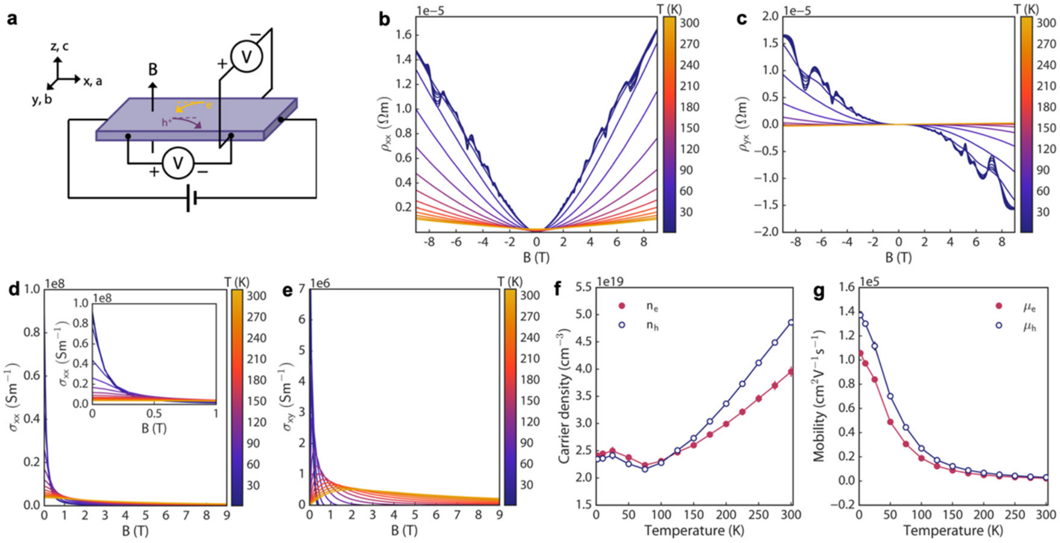

Figure S3**. Carrier concentration and mobility. a** Schematic diagram of the electrical transport measurement in the six-probe geometry. e- and h+ denote electrons and holes,

respectively. Longitudinal and transverse resistivities and conductivities **b** ρ_{xx}, **c** ρ_{yx}, **d** σ_{xx}, and **e** σ_{xy} as functions of magnetic field at different temperatures. **f** Carrier concentration and **g** mobility of electrons and holes resulting from the two-band model fitting.

To experimentally validate the prediction of a quantized thermoelectric Hall effect requires information about the carrier concentration and mobility. To extract this information, we carried out a delicate electrical transport measurement with the ETO of the PPMS. The measurement was done using a standard six-probe geometry, schematically shown in Figure S3a. With the symmetric probe configuration, the measured longitudinal resistivity ρ_{xx} is symmetric with respect to the applied magnetic field, while the transverse resistivity ρ_{yx} is antisymmetric, as shown in Figure S3b and c. In both ρ_{xx} and ρ_{yx}, strong Shubnikov-de Haas (SdH) oscillations can be observed at low temperatures. The oscillation is preserved up to 25K, indicating high-quality crystallization in our sample, as the temperature damping effect would otherwise eliminate the quantum oscillation at this relatively high temperature.

Because the contacts on the sample were made manually with silver epoxy, the measured data exhibit slight asymmetry due to slight misalignment of the contacts. To eliminate the effect of the contact misalignment, we averaged the ρ_{xx} and ρ_{yx} using the equations listed below:

$$\rho_{xx}(B) = \frac{\rho_{xx}(+B) + \rho_{xx}(-B)}{2}, \quad \rho_{yx}(B) = \frac{\rho_{yx}(+B) - \rho_{yx}(-B)}{2}. \qquad \text{(S1)}$$

Then we calculated the longitudinal and transverse conductivities σ_{xx} and σ_{xy} using the following equations:

$$\sigma_{xx} = \frac{\rho_{xx}}{\rho_{xx}^2 + \rho_{xy}^2}, \quad \sigma_{xy} = -\frac{\rho_{xy}}{\rho_{xx}^2 + \rho_{xy}^2} = \frac{\rho_{yx}}{\rho_{xx}^2 + \rho_{yx}^2}. \tag{S2}$$

The field dependence of σ_{xx} and σ_{xy} at various temperatures is shown in Figures S3d and e. To extract the carrier concentration and mobility, we simultaneously fit the σ_{xx} and σ_{xy} data as functions of B using a two-band model defined by:

$$\begin{aligned} \sigma_{xx} &= \frac{n_e \mu_e e}{1+(\mu_e B)^2} + \frac{n_h \mu_h e}{1+(\mu_h B)^2} \\ \sigma_{xy} &= \left[n_h \mu_h^2 \frac{1}{1+(\mu_h B)^2} - n_e \mu_e^2 \frac{1}{1+(\mu_e B)^2} \right] eB \end{aligned}, \tag{S3}$$

where n_e and n_h denote the electron and hole carrier densities, μ_e and μ_h are the corresponding mobilities, and e is the elementary charge. We thereby extract the electron and hole carrier densities and mobilities as functions of temperature, as shown in Figures S3f and g. The electron and hole concentrations are nearly compensated at low temperatures. This proves the origin of the giant magnetoresistance.

4. Analysis of Quantum Oscillation

Since the carrier pockets analysis based on quantum oscillation can be influenced by the choice of background of magnetoresistance (MR), we adopted three independent methods using 1) background-free curvature approach (Figure S4), 2) a T=25K data without quantum oscillation as background (Figure S5), and 3) a fitted background to a linear-quadratic function (Figure S6), all of which lead to a consistent conclusion of the existence of a low frequency carrier pocket $F_\alpha = 2.3T \sim 4T$. This enables the possibility that the carrier pockets of W2 Weyl point can indeed reach the desired n=0 LL.

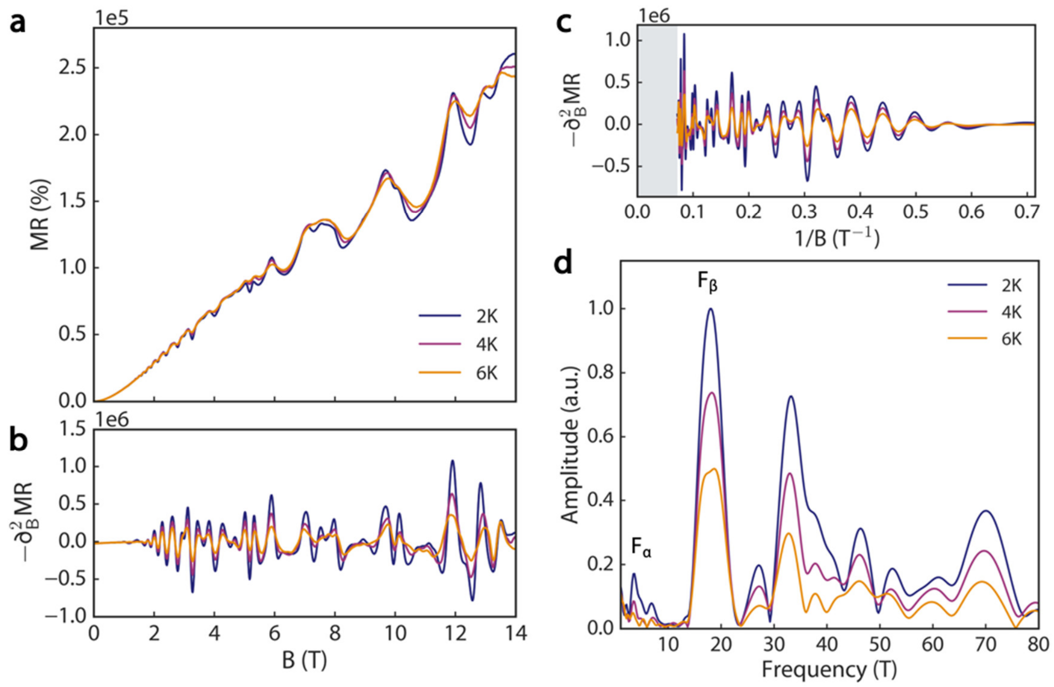

Figure S4. **MR analysis using curvature-based background subtraction.** In this approach, a second-order derivative against magnetic field *B* is taken to the MR data, where all linear, constant, and quadratic terms will be automatically wiped out without need to manually choosing background. Although this method is seldom used, this may offer an alternative but strong approach for MR analysis. **a** MR data up to *B*=14T, at *T*=2K, 4K and 6K. $\partial_B^2 MR$ as a function of **b** *B* and **c** 1/*B*. **d** Fourier transform of (c), showing the two carrier pockets $F_\alpha = 3.8T$ and $F_\beta = 18T$.

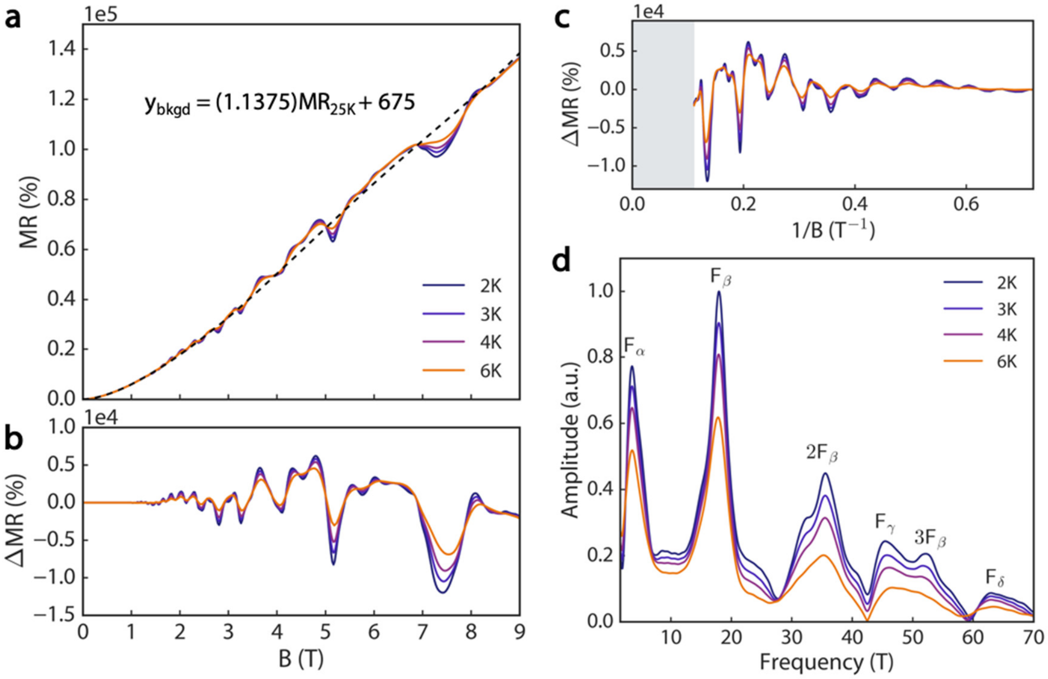

Figure S5. **MR analysis using *T*=25K data as background.** In this approach, since the quantum oscillation fully disappears at *T*=25K, we can use the MR at *T*=25K as the background. **a** The low-temperature MR data and the background using linearly transformed MR at 25K (black dashed line). The ΔMR after background subtraction in terms of **b** *B* and **c** *1/B*. **d** The Fourier transform of (c), showing the two carrier pockets $F_\alpha = 4T$ and $F_\beta = 18T$.

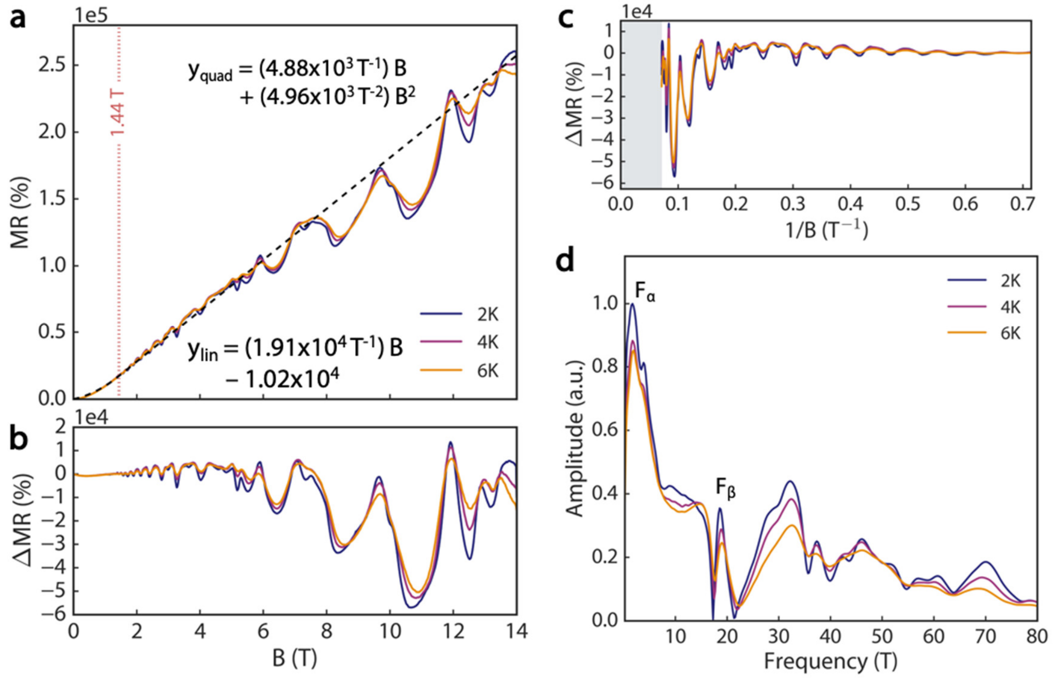

Figure S6. **MR analysis using polynomial fitting-based background subtraction.** In this approach, an optimal threshold B_0 is obtained by curve-fitting, which divides the measurement into a quadratic ($B < B_0$) and linear ($B > B_0$) regime, with their magnitudes and slopes matched at B_0 . **a** The low-temperature MR data and a fitted linear-quadratic function as background (black dashed line). The red vertical dashed line indicates the fitted value $B_0 = 1.44\text{T}$. The ΔMR after background subtraction in terms of **b** *B* and **c** *1/B*. **d** The Fourier transform of **c**, showing the two carrier pockets $F_\alpha = 2.3T$ and $F_\beta = 19T$. Despite a small quantitative difference, the existence of the low-frequency carrier pocket is confirmed.

5. Landau Level and Quantum Limit

The quantized thermoelectric Hall effect considered in this work is theoretically predicted to exist in the quantum limit of Dirac/Weyl semimetals[1, 2]. Therefore, to examine the validity of the theoretical prediction, we first verify that the quantum limit condition is satisfied by Weyl fermions in our TaP sample. To do this, we performed a thorough analysis of the quantum oscillations observed in the electrical transport measurement, as shown in Figure 1 in the main text and discussed in detail in the previous section. The quantum oscillation data ΔMR shown in Figure 1d of the main text was obtained by subtracting a smooth background from the magnetoresistance (MR) data, Figure 1c, where MR is defined according to:

$$MR = \frac{\rho_{xx}(B) - \rho_{xx}(B = 0\mathrm{T})}{\rho_{xx}(B = 0\mathrm{T})} \times 100\%. \qquad \text{(S4)}$$

From the fast Fourier transform (FFT) analysis depicted in Figure 1e, we observe four noticeable oscillation frequencies: $F_\alpha = 4\mathrm{T}$, $F_\beta = 18\mathrm{T}$, $F_\gamma = 46\mathrm{T}$, and $F_\delta = 64\mathrm{T}$. After performing a standard signal filtering process by performing inverse FFT to the two relatively low frequencies of 4T and 18T individually, we isolate the two oscillation components from the pristine data and determine the corresponding Landau levels (LLs) by assigning an integer (half-integer) value to the oscillation maxima (minima), as shown in Figure 1f. From the LL index fan, we conclude that in our TaP sample, the α Fermi pocket corresponding to the 4T frequency is in the n=0 LL at our maximum field of B=9T, whereas the β Fermi pocket corresponding to the 18T frequency is in the n=2 LL. Specifically, the α Fermi pocket enters the quantum limit (lowest LL)

approximately at 3.8T, and the β Fermi pocket will reach the quantum limit at an approximate field of 16T. The linear fitting of the LL index as a function of 1/B yields intercepts of -0.037 and 0.065 for α and β, respectively. Both are in the range of -1/8 to 1/8, proving the bands in the α and β Fermi pockets are topologically non-trivial and thus Weyl cones are present[S3]. From this, we can further conclude that the Weyl fermions in the smallest Fermi pocket of TaP are well within the quantum limit at our maximum applied field, whereas the Weyl fermions in the second smallest Fermi pocket are nearing the onset of the quantum limit.

6. Data Analysis for Thermoelectric Measurement

Figure S7a schematically shows the principle behind the thermal transport measurement in the diagonal offset probe geometry. Using the TTO of the PPMS, the heater on the left end of the thinned-down crystal and heat sink on the right establish a continuous heat flow along the *a* or *b* axis (*a* and *b* are equivalent for this tetragonal system), as shown in Figure S7. The thermal conductivity is directly calculated by the PPMS using the applied heater power, the resulting temperature difference ΔT detected between the two thermometers, and the sample dimension. The voltage drop ΔV between the two thermometers is monitored simultaneously, which yields the Seebeck signals by calculation of $-\Delta V/\Delta T$. A magnetic field was applied along the *c* axis for detecting the proposed quantized thermoelectric Hall effect. Figure S7b shows the temperature dependence of thermal conductivity of TaP at 9T and -9T. From this plot, we note that the thermal conductivities at positive and negative magnetic fields have a very slight difference. This indicates that the thermoelectric Hall effect (the transverse movement of thermal electrons in the presence of a magnetic field) provides a negligible but

observable heat flow along the transverse direction. To extract the longitudinal thermal conductivity from the measured thermal conductivity, we use the following equations:

$$\rho_{th,xx}(B) = \frac{\kappa_{\text{meas}}(+B) + \kappa_{\text{meas}}(-B)}{2\kappa_{\text{meas}}(+B)_{\text{meas}}\kappa(-B)},\ \rho_{th,yx}(B) = \frac{\kappa_{\text{meas}}(+B) - \kappa_{\text{meas}}(-B)}{2\kappa_{\text{meas}}(+B)\kappa_{\text{meas}}(-B)} \times \frac{L}{W}, \tag{S5}$$

and

$$\kappa_{xx} = \frac{\rho_{th,xx}}{\rho_{th,xx}^2 + \rho_{th,yx}^2}, \tag{S6}$$

where L and W represent the length-wise and the width-wise separation between the two thermometers. Figure S7c displays the obtained longitudinal thermal conductivity κ_{xx} as a function of temperature at different magnetic fields. From the inset of Figure S7c, we see that the applied magnetic field gradually suppresses the longitudinal thermal conductivity. This phenomenon is consistent with the giant magnetoresistance, as both originate from the greatly elevated electron scattering induced by the magnetic field. The magnitude of the thermal conductivity of TaP is very large compared to most materials, which explains the importance of thinning the sample prior to measurement. The Seebeck signals at 0T, 9T and -9T are plotted in Figure S7d, from which giant magnetic field-induced Seebeck signals can be observed at 9T and -9T. The data for 9T and -9T are asymmetrical due to the mutual presence of longitudinal and transverse Seebeck signals. We use the following equations to calculate the longitudinal and transverse Seebeck coefficients S_{xx} and S_{yx}:

$$S_{xx}(B) = \frac{S_{\text{meas}}(+B) + S_{\text{meas}}(-B)}{2},\ S_{yx}(B) = \frac{S_{\text{meas}}(+B) - S_{\text{meas}}(-B)}{2} \times \frac{L}{W}. \tag{S7}$$

The temperature dependence of S_{xx} and S_{yx} collected at different magnetic fields is presented in Figures S7e and f. It is obvious that the applied magnetic fields induce

giant Seebeck coefficients along both longitudinal and transverse directions. The longitudinal Seebeck coefficient S_{xx} does not appear to saturate with increasing field up to the highest measured field of 9T. By contrast, S_{yx} tends to saturate at high magnetic fields. Another novel behavior in S_{xx} and S_{yx} is the presence of a double-peak feature around T=40K. We provide a clear explanation of this feature in the main text.

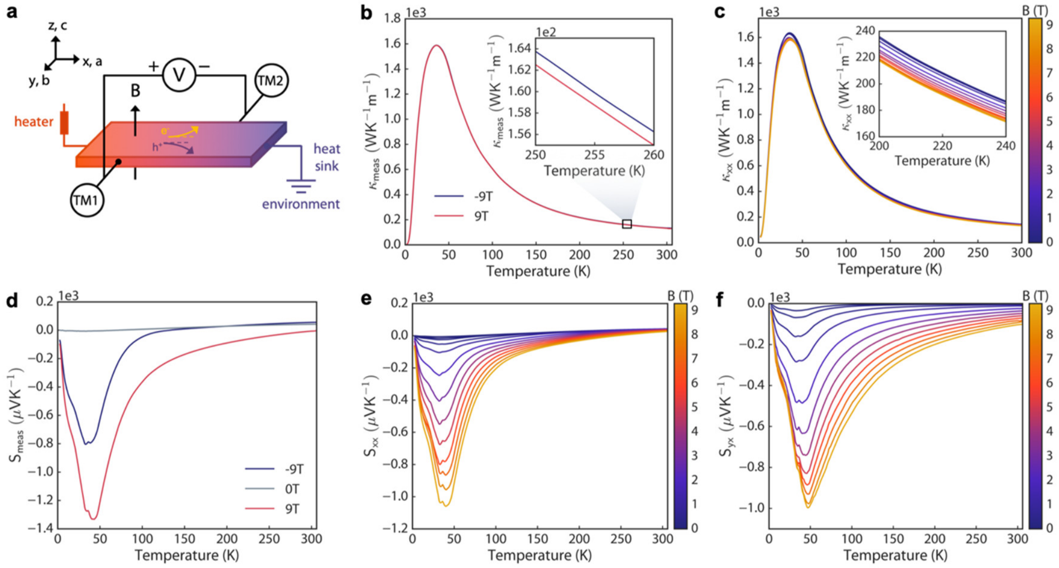

Figure S7. **Data analysis in thermoelectric measurements. a** Schematic diagram of the thermal transport measurement. TM1 and TM2 represent thermometer 1 and 2. The temperature difference between the short ends of the sample is represented by the color gradient from red (high) to blue (low). e- and h+ denote electrons and holes, respectively. **b** Thermal conductivities of TaP at 9T and -9T. **c** Longitudinal thermal conductivity of TaP as a function of temperature at various fields. **d** Measured Seebeck signals at 0T, 9T and -9T for the diagonal offset probe geometry. **e** Longitudinal and **f** transverse Seebeck coefficients S_{xx} and S_{yx} as functions of temperature at different magnetic fields.

After performing the thermal transport measurement at a certain temperature, a subsequent electrical transport measurement at the same temperature is made with the

TTO. The inset of Figure S8a shows the schematic diagram for the electrical transport measurement in the diagonal offset geometry. In the presence of a magnetic field, the system applies an electrical current along the *a* or *b* axis, and the voltmeter between the diagonal offset probes detects the voltage drop which contains both longitudinal and transverse components. The longitudinal resistivity ρ_{xx} and the transverse resistivity (also called Hall resistivity) ρ_{yx} are separated using the following equations:

$$\rho_{xx}(B) = \frac{\rho_{\text{meas}}(+B) + \rho_{\text{meas}}(-B)}{2}, \quad \rho_{yx}(B) = \frac{\rho_{\text{meas}}(+B) - \rho_{\text{meas}}(-B)}{2} \times \frac{L}{W}. \qquad \text{(S8)}$$

Figure S8a displays the measured resistivity at 0T, 9T and -9T. The disagreement between the 9T and -9T data is evidence of the mutual presence of the longitudinal and transverse resistivities ρ_{xx} and ρ_{yx}. After separating ρ_{xx} and ρ_{yx} using Eq. (S8), as shown in Figures S8b and c, we then calculated the figure of merit zT according to:

$$zT = \frac{S_{xx}^2 T}{\rho_{xx}\kappa_{xx}}. \qquad \text{(S9)}$$

From the plot of zT in Figure S8d, we note that, although the power factor (shown in Figure 2f in the main text) is record-breaking in magnitude, the zT does not attain a very high value due to the significant thermal conductivity.

It should be noted that the giant magnetic field-induced Seebeck coefficients cannot be observed in the case of $B \| a \| j_Q$, which is evidenced by comparison of two geometries in Figures S8e and f. This indicates that the giant magnetic field-induced longitudinal and transverse Seebeck coefficients in the case of $B \| c \perp j_Q$ originate from the quantized protection of the thermoelectric Hall Effect.

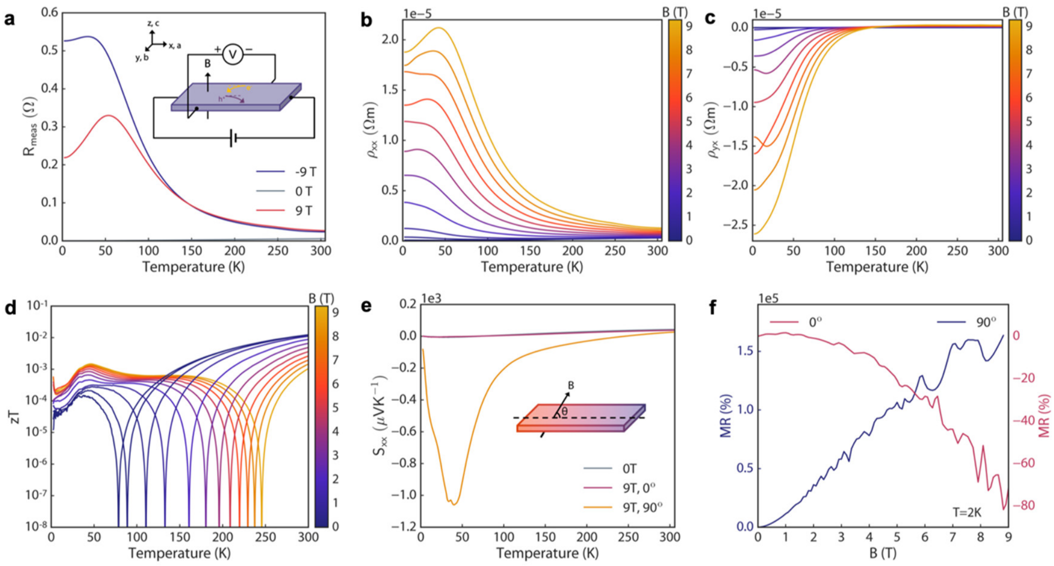

Figure S8. **Comparison between perpendicular and parallel configurations. a** Measured resistivities of TaP at 0T, 9T and -9T for the diagonal offset probe geometry. Inset: Schematic diagram of the electrical transport measurement. Longitudinal and transverse resistivities **b** ρ_{xx}, **c** ρ_{yx}, and **d** zT as functions of temperature at different magnetic fields. Comparison of the $B \| a \| j_Q$ and $B \| c \perp j_Q$ geometries for **e** S_{xx} and **f** MR. The giant Seebeck coefficients were not observed in the $B \| a \| j_Q$ case.

To summarize the phase relations of various thermoelectric quantities, we plot the resistivity, thermopower, thermal conductivity, and thermoelectric Hall conductivity in both longitudinal and transverse directions and highlight their phase relations, as done in Figures S9-S13.

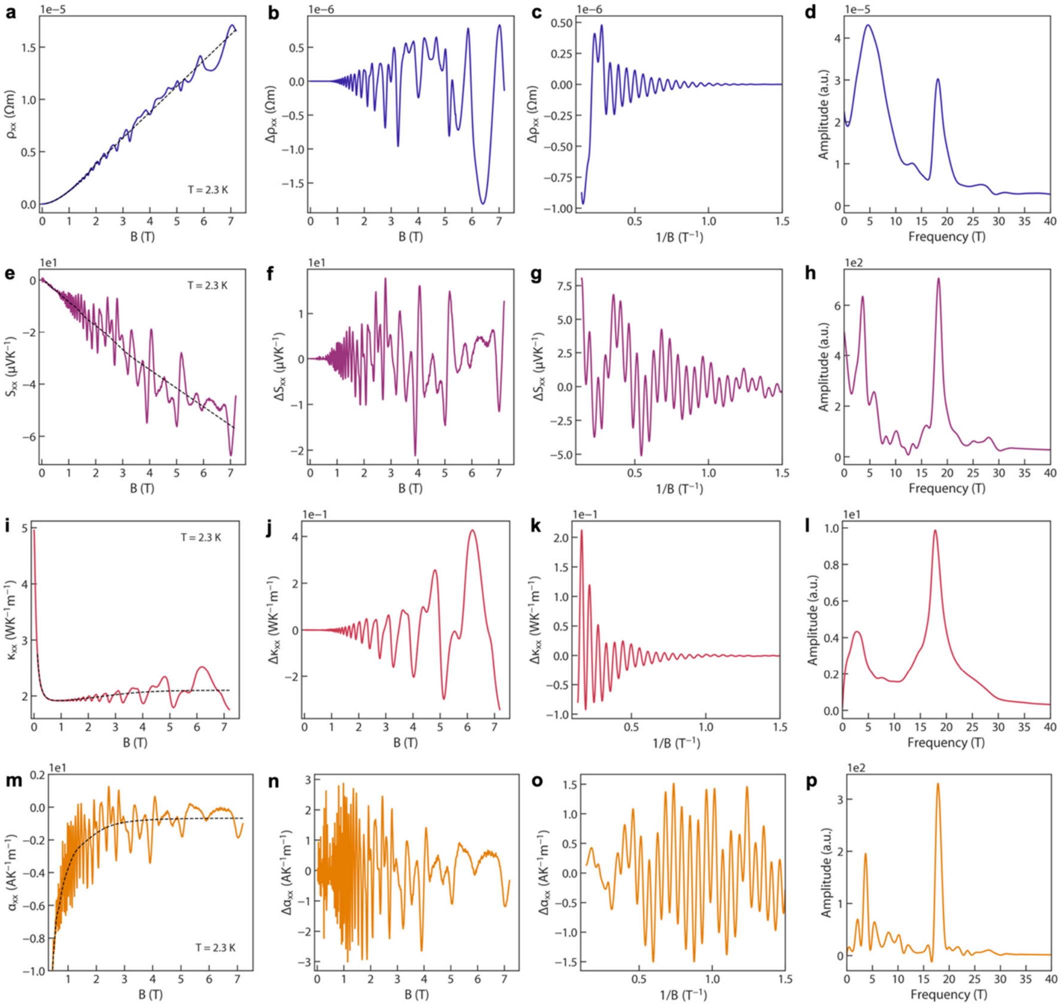

Figure S9. **Quantum oscillation of longitudinal thermoelectric properties at base temperature. a-d** Longitudinal resistivity ρ_{xx}. **a** ρ_{xx} vs B, **b** the background-subtracted part $\Delta\rho_{xx}$ vs B, **c** $\Delta\rho_{xx}$ vs $1/B$, and **d** the Fourier transform of **c**. **e-h** Longitudinal thermopower S_{xx}. **e** S_{xx} vs B, **f** the background-subtracted part ΔS_{xx} vs B, **g** ΔS_{xx} vs $1/B$, and **h** the Fourier transform of **g**. **i-l** Longitudinal thermal conductivity κ_{xx}. **i** κ_{xx} vs B, **j** the background-subtracted part $\Delta\kappa_{xx}$ vs B, **k** $\Delta\kappa_{xx}$ vs $1/B$, and **l** the Fourier transform of **k**. **m-p** Longitudinal thermoelectric conductivity α_{xx}. **m** α_{xx} vs B, **n** the background-subtracted part $\Delta\alpha_{xx}$ vs B, **o** $\Delta\alpha_{xx}$ vs $1/B$, and **p** the Fourier transform of **o**.

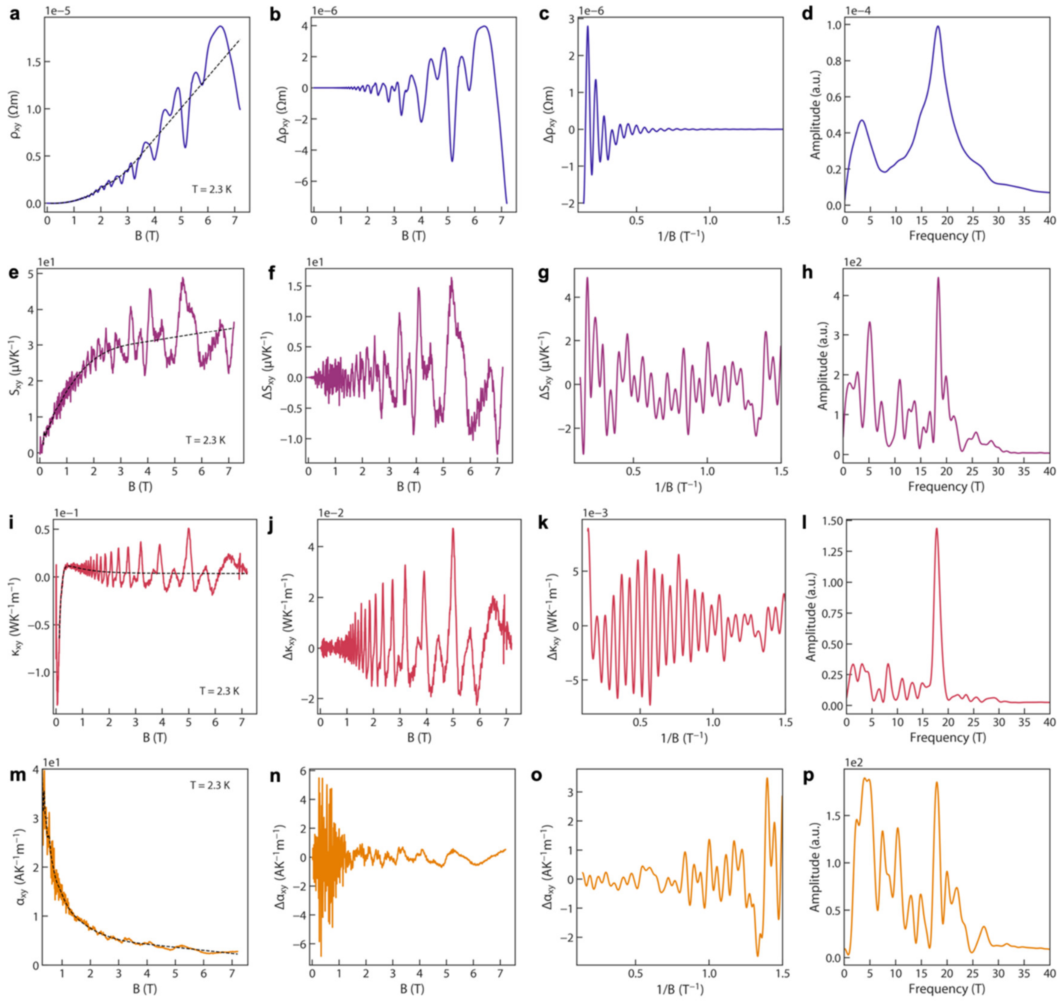

Figure S10. **Quantum oscillation of transverse thermoelectric properties at base temperature. a-d** Transverse resistivity ρ_{xy}. **a** ρ_{xy} vs *B*, **b** the background-subtracted part $\Delta\rho_{xy}$ vs *B*, **c** $\Delta\rho_{xy}$ vs $1/B$, and **d** the Fourier transform of **c**. **e-h** Transverse thermopower S_{xy}. **e** S_{xy} vs *B*, **f** the background-subtracted part ΔS_{xy} vs *B*, **g** ΔS_{xy} vs $1/B$, and **h** the Fourier transform of **g**. **i-l** Transverse thermal conductivity κ_{xy}. **i** κ_{xy} vs *B*, **j** the background-subtracted part $\Delta\kappa_{xy}$ vs *B*, **k** $\Delta\kappa_{xy}$ vs $1/B$, and **l** the Fourier transform of **k**. **m-p** Transverse thermoelectric conductivity α_{xy}. **m** α_{xy} vs *B*, **n** the background-subtracted part $\Delta\alpha_{xy}$ vs *B*, **o** $\Delta\alpha_{xy}$ vs $1/B$, and **p** the Fourier transform of **o**.

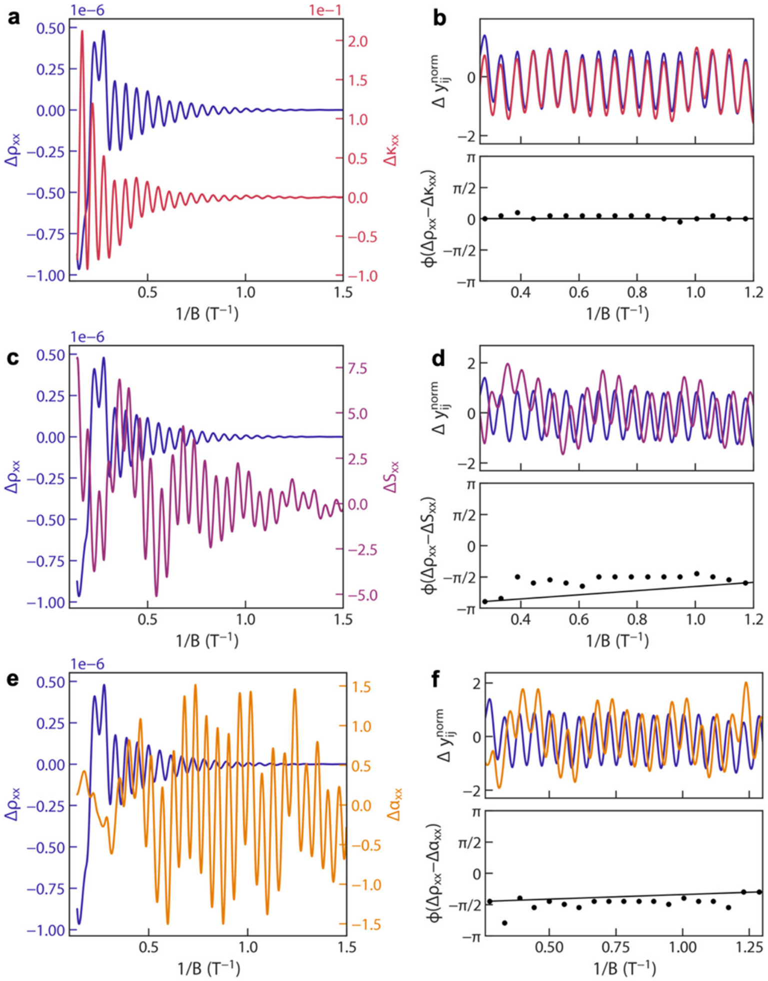

Figure S11. **Phase relations of longitudinal thermoelectric properties. a-b** Resistivity vs thermopower. **a** Quantum oscillation of background-subtracted longitudinal resistivity $\Delta\rho_{xx}$ against the background-subtracted longitudinal thermopower ΔS_{xx}, and **b** the corresponding amplitude-normalized curves highlighting the phase relation between $\Delta\rho_{xx}$ and ΔS_{xx} (upper figure), and the phase difference between $\Delta\rho_{xx}$ and ΔS_{xx} (lower figure) as a function of $1/B$. **c-d** Resistivity vs thermal conductivity. **c** Quantum oscillation of background-subtracted longitudinal resistivity $\Delta\rho_{xx}$ against the background-subtracted longitudinal thermal conductivity $\Delta\kappa_{xx}$, and **d** the corresponding amplitude-normalized curves highlighting the phase relation between $\Delta\rho_{xx}$ and $\Delta\kappa_{xx}$ (upper figure), and the phase difference between $\Delta\rho_{xx}$

and $\Delta\kappa_{xx}$ (lower figure) as a function of $1/B$. **e-f** Resistivity vs thermoelectric conductivity. **e** Quantum oscillation of background-subtracted longitudinal resistivity $\Delta\rho_{xx}$ against the background-subtracted longitudinal thermoelectric conductivity $\Delta\alpha_{xx}$, and **f** the corresponding amplitude-normalized curves highlighting the phase relation between $\Delta\rho_{xx}$ and $\Delta\alpha_{xx}$ (upper figure), and the phase difference between $\Delta\rho_{xx}$ and $\Delta\alpha_{xx}$ (lower figure) as a function of $1/B$.

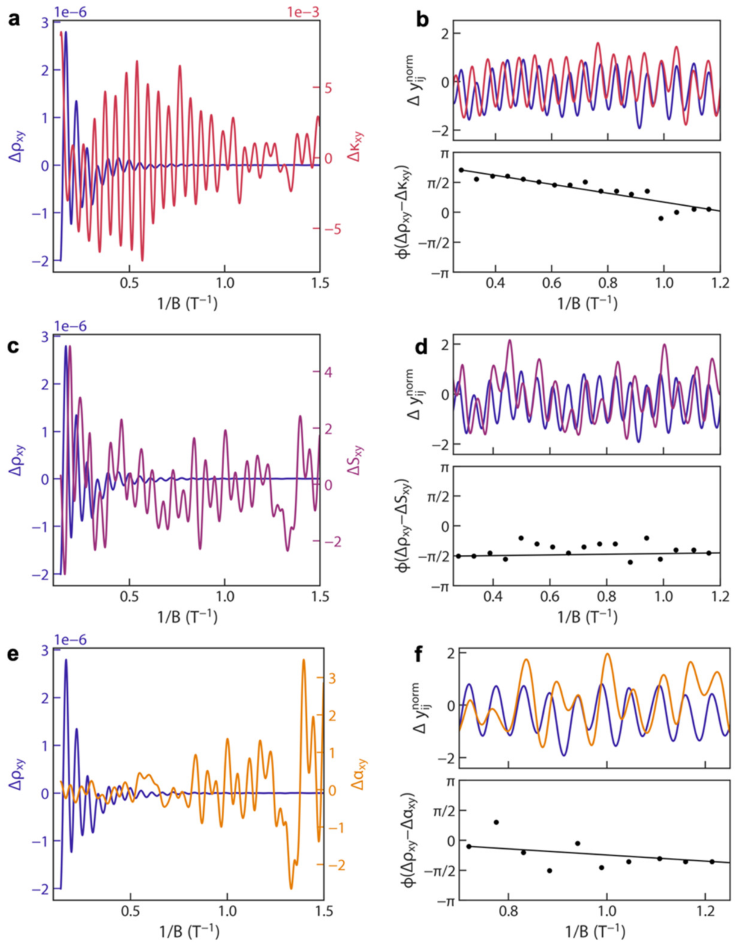

Figure S12. **Phase relations of transverse thermoelectric properties. a-b** Resistivity vs thermopower. **a** Quantum oscillation of background-subtracted transverse resistivity $\Delta\rho_{xy}$ against the background-subtracted transverse thermopower ΔS_{xy}, and **b** the corresponding

amplitude-normalized curves highlighting the phase relation between $\Delta\rho_{xy}$ and ΔS_{xy} (upper figure), and the phase difference between $\Delta\rho_{xy}$ and ΔS_{xy} (lower figure) as a function of $1/B$. **c-d** Resistivity vs thermal conductivity. **c** Quantum oscillation of background-subtracted transverse resistivity $\Delta\rho_{xy}$ against the background-subtracted transverse thermal conductivity $\Delta\kappa_{xy}$, and **d** the corresponding amplitude-normalized curves highlighting the phase relation between $\Delta\rho_{xy}$ and $\Delta\kappa_{xy}$ (upper figure), and the phase difference between $\Delta\rho_{xy}$ and $\Delta\kappa_{xy}$ (lower figure) as a function of $1/B$. **e-f** Resistivity vs thermoelectric conductivity. **e** Quantum oscillation of background-subtracted transverse resistivity $\Delta\rho_{xy}$ against the background-subtracted transverse thermoelectric conductivity $\Delta\alpha_{xy}$, and **f** the corresponding amplitude-normalized curves highlighting the phase relation between $\Delta\rho_{xy}$ and $\Delta\alpha_{xy}$ (upper figure), and the phase difference between $\Delta\rho_{xy}$ and $\Delta\alpha_{xy}$ (lower figure) as a function of $1/B$.

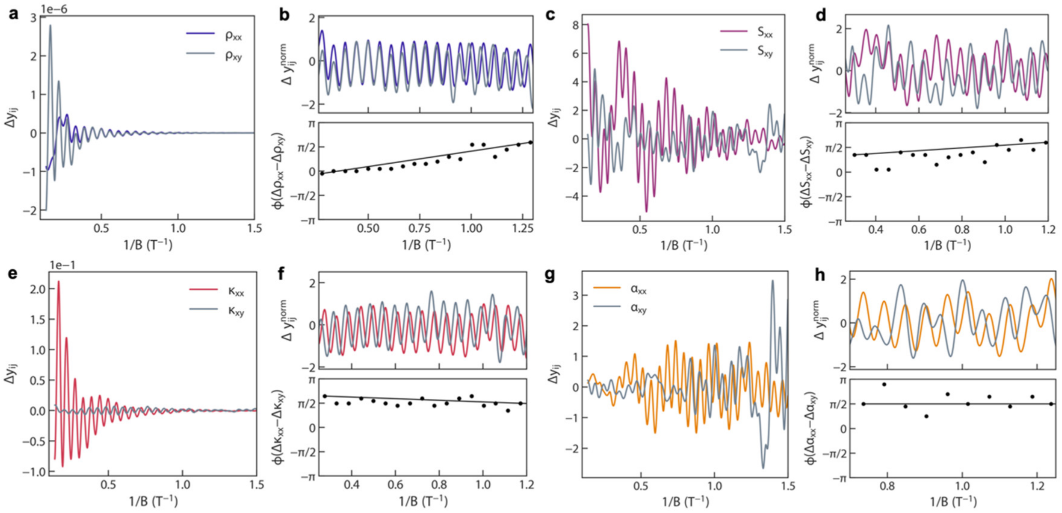

Figure S13. **Phase relations between longitudinal and transverse thermoelectric properties.** **a-b** Phase relation between $\Delta\rho_{xx}$ and $\Delta\rho_{xy}$. **c-d** Phase relation between ΔS_{xx} and ΔS_{xy}. **e-f** Phase relation between $\Delta\kappa_{xx}$ and $\Delta\kappa_{xy}$. **g-h** Phase relation between $\Delta\alpha_{xx}$ and $\Delta\alpha_{xy}$.

7. Thermoelectric Hall Conductivity up to 9T

To validate the quantized thermoelectric Hall effect, particularly the quantized plateau of the thermoelectric Hall coefficient α_{xy} in the high magnetic field limit, we calculated α_{xy} using the following equation:

$$\alpha_{xy} = \frac{-\rho_{xy}S_{xx} + \rho_{xx}S_{xy}}{\rho_{xx}^2 + \rho_{xy}^2} = \frac{\rho_{yx}S_{xx} - \rho_{xx}S_{yx}}{\rho_{xx}^2 + \rho_{yx}^2}. \quad \text{(S10)}$$

To obtain α_{xy} as a function of magnetic field for different temperatures, we replotted S_{xx} and S_{yx} from Figures S7e and f, and ρ_{xx} and ρ_{yx} from Figures S8b and c, as functions of magnetic field, as shown in Figures 2d and 3a in the main text and Figures S14a and b in the SI. The resulting α_{xy} calculated with Eq. (S10) is displayed in Figure 3b in the main text.

To extract the values of effective Fermi velocity v_F^{eff} and chemical potential μ, as well as identify the quantized value of α_{xy}/T approached at very large fields, we fit our low-temperature α_{xy} data up to T=10K using the general expression of α_{xy} in the dissipationless limit[2] (Eq. (3) of the main text):

$$\alpha_{xy} = \frac{eN_f}{2\pi\hbar}\sum_{n=0}^{\infty}{}' \int_0^{\infty} \frac{dk_z}{\pi}\left[s\left(\frac{\varepsilon_n^0(k_z)-\mu}{k_BT}\right) + s\left(\frac{\varepsilon_n^0(k_z)+\mu}{k_BT}\right)\right]. \quad \text{(S11)}$$

where the notation $\sum_{n=0}^{\infty}{}'$ is used to mean that an extra factor of 1/2 multiplies the n=0 term of the sum; N_f equals the number of Weyl points, and $\varepsilon_n^0(k_z)$ denote the Landau level energies:

$$\varepsilon_n^0(k_z) = \mathrm{sgn(n)} v_F \sqrt{2e\hbar B|n| + \hbar^2 k_z^2}\,. \tag{S12}$$

and v_F is treated as v_F^{eff}. The function $s(x)$ is the entropy per carrier, given by

$$s(x) = -k_B\left[n_F(x)\ln n_F(x) + \left(1-n_F(x)\right)\ln\left(1-n_F(x)\right)\right]. \tag{S13}$$

where $n_F(x) = \left(1+e^{\beta x}\right)^{-1}$ is the Fermi-Dirac distribution. The data to be fitted using Eq. (S11) is shown in Figure S14c, and we extrapolate the fitted function to even larger magnetic fields, revealing we are near the onset of the quantized limit. The value of α_{xy}/T approached in this limit is $\sim 0.4\mathrm{AK^{-2}m^{-1}}$. The corresponding fitted parameters are given in Figures S14d and e.

To verify this fit, we additionally fit our low-temperature data up to T=50K using the expression for α_{xy}/T which also includes a finite scattering time τ and is thus a more expressive form for data with weak scattering present[2]:

$$\alpha_{xy} = \frac{N_f}{18}\frac{e^2 k_B^2 T v_F \tau^2 B}{\hbar^3}\frac{1+3\omega_c^2(E_F)\tau^2}{\left(1+\omega_c^2(E_F)\tau^2\right)^2}. \tag{S14}$$

where the cyclotron frequency ω_c is given by

$$\omega_c(\varepsilon) = \frac{eBv_F^2}{\varepsilon}. \tag{S15}$$

and once more, v_F is treated as v_F^{eff}. This fit is shown in Figure S14f with the corresponding fitted parameters shown in Figures S14g and h, which are in good agreement with those of the previous fit.

Similarly, we fit our high-temperature data, T>50K, in the limit of weak scattering using

$$\alpha_{xy} = \frac{N_f e^2 k_B^2 T v_F \tau^2 B}{6\pi^2 \hbar^3} \int_{-\infty}^{+\infty} dx \frac{x^4 e^x}{(1+e^x)^2} \frac{1}{x^2 + \omega_c^2 (k_B T) \tau^2}. \qquad \text{(S16)}$$

which is shown in Figure S14i with corresponding fitted parameters plotted in Figures S14j and k.

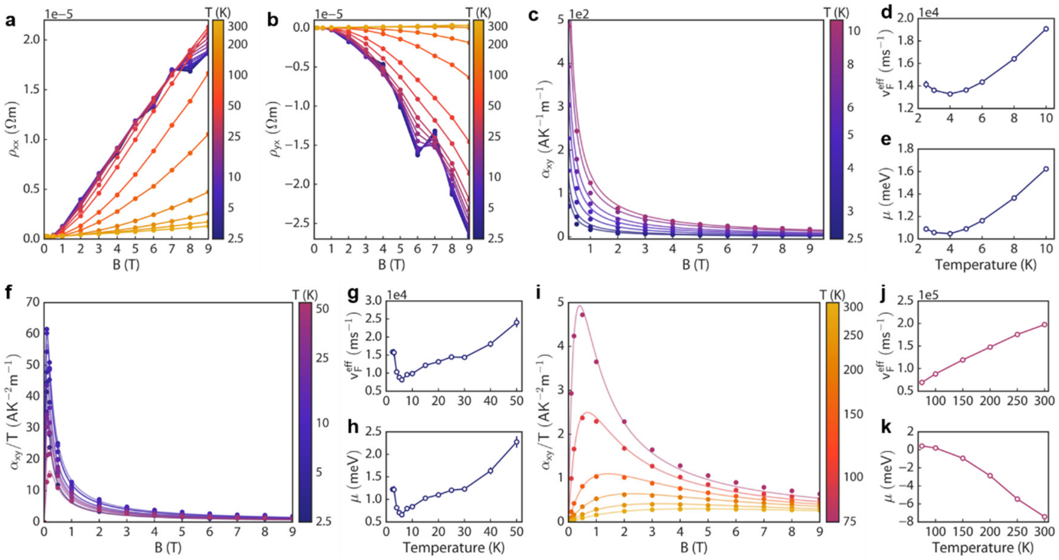

Figure S14. **Data analysis of thermoelectric Hall conductivity.** Longitudinal and transverse resistivities **a** ρ_{xx} and **b** ρ_{yx} as functions of magnetic field at different temperatures. **c** Thermoelectric Hall conductivity α_{xy} as a function of magnetic field at different temperatures. The solids lines are fitted curves using Eq. (S11) (low-temperature dissipationless limit), shown as solid lines. **d-e** Effective Fermi velocity v_F^{eff} and chemical potential μ obtained from the fitting in **c** using on Eq. (S11). **f** α_{xy}/T fitted using Eq. (S14), where the corresponding fitting parameters are shown in **g** and **h**. At higher temperature, **i** the α_{xy}/T is fitted with Eq. (S16),

and the corresponding fitted parameters are shown in **j** and **k**. It can be seen that there is a general quantitative agreement using different fitting equations.

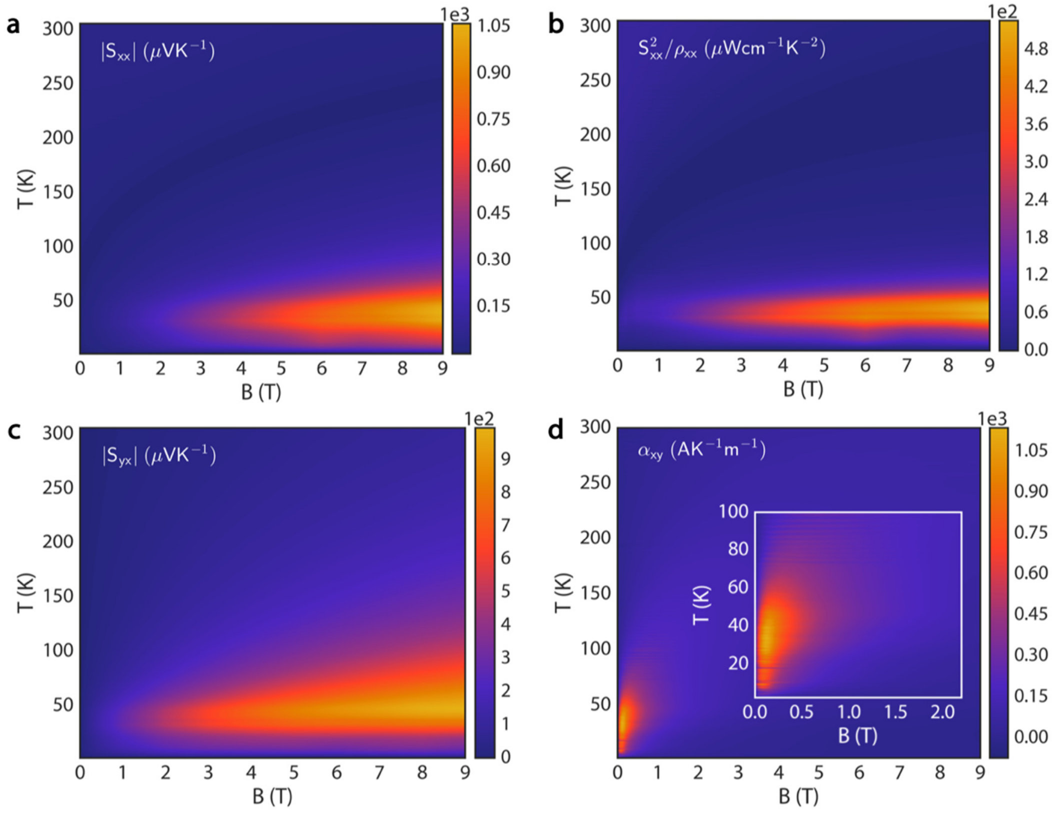

Figure S15. **The 2D contour plots of thermoelectric performance,** including **a** $\left|S_{xx}\right|$, **b** S_{xx}^2/ρ_{xx}, **c** $\left|S_{yx}\right|$, and **d** α_{xy} showing comprehensive data collection from B=0T to 9T, and from T=2K to 300K.

8. Low-temperature Thermoelectric Measurements up to 14T

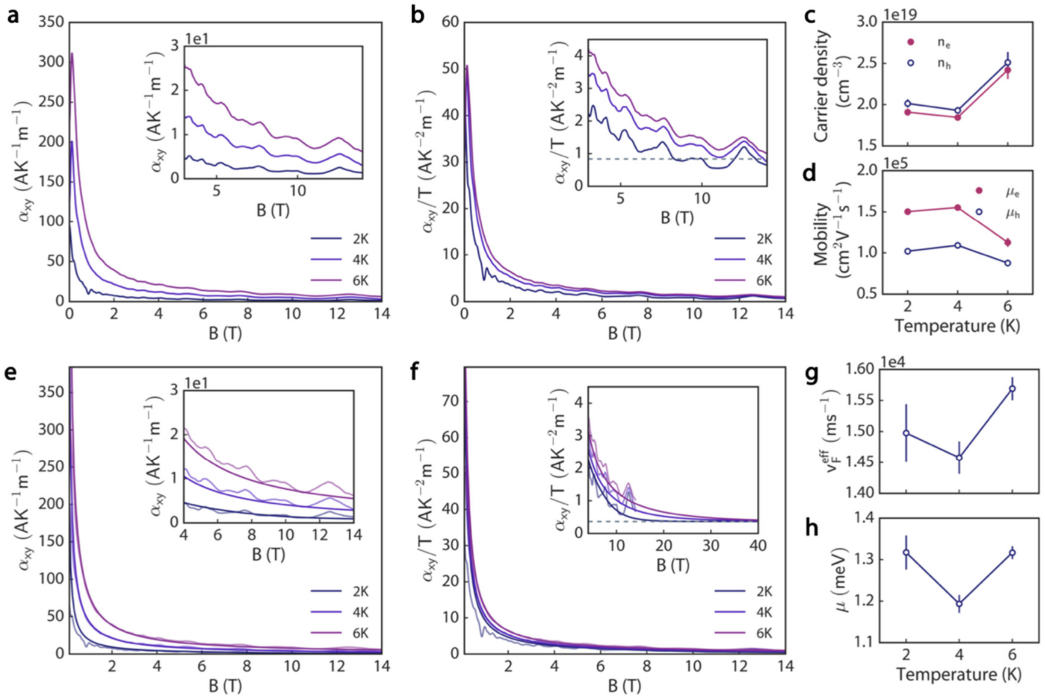

Figure S16. **Data analysis of low-temperature, high-field thermoelectric Hall conductivity.** The thermoelectric Hall conductivity **a** α_{xy} and **b** the ratio α_{xy}/T at T=2K, 4K and 6K up to 14T. We can see a clear flattening trend that persists beyond 9T whereby the three different temperature curves collapse into one. The **c** carried density and the **d** mobility obtained by fitting. **e-f** Identical α_{xy} and α_{xy}/T data, overlaid on top of the fitting; the universality can be seen by extending to high magnetic field, resulting in the universal value $\alpha_{xy}/T = 0.37\mathrm{AK^{-2}m^{-1}}$, consistent with the separate 9T data. **g-h** The effective Fermi velocity and chemical potential are also in excellent agreement with the 9T data.

9. Dominant Thermoelectric Hall Contribution to Longitudinal Thermoelectric Performance at Low Temperatures

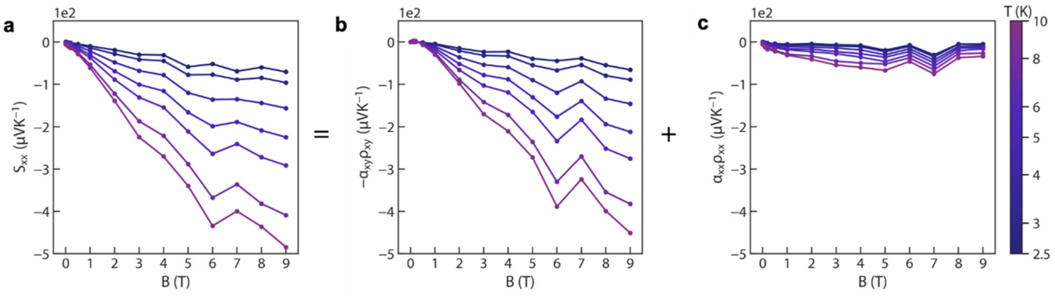

Figure S17. **Dominant contribution of longitudinal thermopower** S_{xx} **from the transverse thermoelectric Hall conductivity** α_{xy} **at low temperatures. a** Total S_{xx} up to 10K as a function of magnetic field separated into **b** a transverse contribution $-\alpha_{xy}\rho_{xy}$ and **c** a longitudinal contribution $+\alpha_{xx}\rho_{xx}$ **c**. All results show that the transverse component dominantly contributes over 90% of the longitudinal thermopower value at low temperatures.

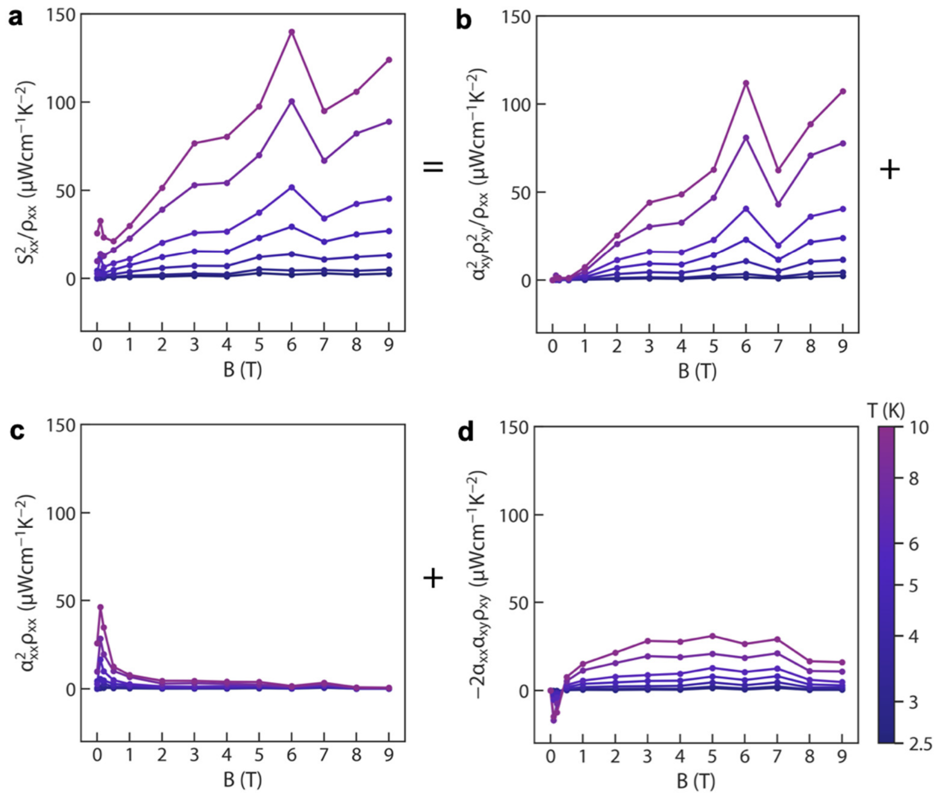

Figure S18. **Dominant contribution of longitudinal power factor S_{xx}^2/ρ_{xx} from the transverse thermoelectric Hall conductivity α_{xy} at low temperatures**. **a** Total S_{xx}^2/ρ_{xx} at low temperatures as a function of magnetic field separated into **b** a transverse component $\alpha_{xy}^2\rho_{xy}^2/\rho_{xx}$, **c** a longitudinal contribution $+\alpha_{xx}^2\rho_{xx}$, and **d** a cross term contribution $-2\alpha_{xy}\alpha_{xx}\rho_{xy}$.

10. X-Ray and Neutron Scattering Measurement Details

Inelastic neutron scattering measurements were performed on the HB1 triple-axis spectrometer at the High-Flux Isotope Reactor at the Oak Ridge National Laboratory. We used a fixed E_f = 14.7 meV with 48’−40’−40’−120’ collimation and Pyrolytic Graphite filters to eliminate higher-harmonic neutrons. Measurements were performed using closed-cycle refrigerators between room temperature and the base temperature 4 K. Inelastic X-ray scattering was performed on the high-energy resolution inelastic x-ray (HERIX) instrument at sector 3-ID beamline of the Advanced Photon Source, Argonne National Laboratory with incident beam energy of 21.657 keV (λ=0.5725Å) and an overall energy resolution of 2.1 meV[4, 5, 6]. Incident beam focused on the sample using toroidal and KB mirror system. FWHM of beam size at sample position was 20 × 20 μm^2 (V × H). The spectrometer was functioning in the horizontal scattering geometry with a horizontally polarized radiation. The scattered beam was analyzed by a diced and spherically curved silicon (18 6 0) analyzers working at backscattering angle. The basic principles of such instrumentations are discussed elsewhere[7, 8].

Measurements of the phonon modes along high-symmetry lines in the Brillouin zone of TaP were performed using both inelastic x-ray scattering and inelastic neutron

scattering. Selected raw intensity spectra along high symmetry direction Γ to Σ are shown in Figure S19 using x-rays (left) and neutrons (right). The spectra were analyzed by a damped harmonic oscillator (DHO) model convoluted with the experimental resolution function to yield the energy and intensity of each mode. These were used to generate a phonon dispersion relation, which can be seen in Figure 5c in the main text, along high symmetry line Z-Γ-Σ. These experimental results serve as a consistency check to support the ab initio calculations performed for the thermal conductivity used in the main text and displayed in Figure 5b.

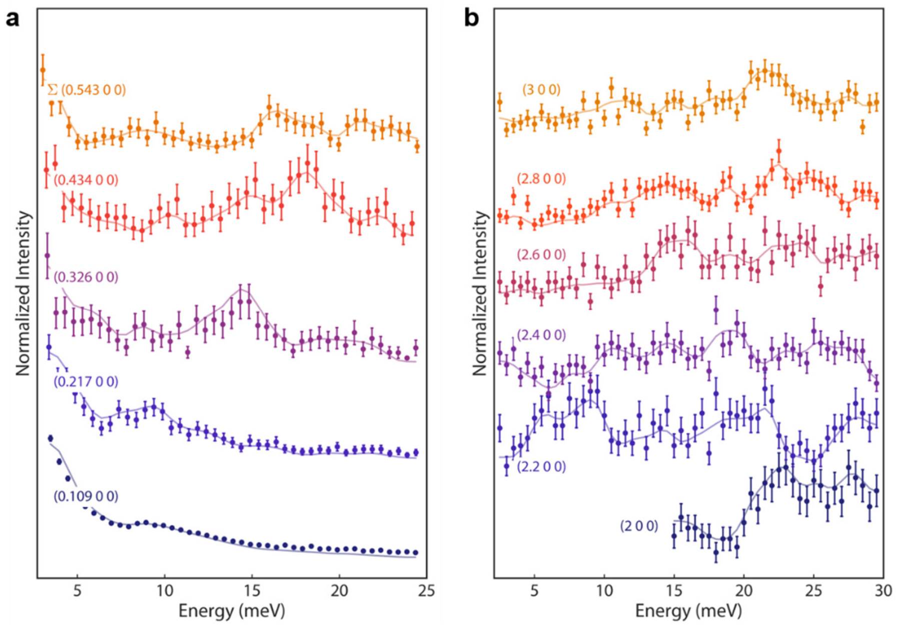

Figure S19**. X-ray and neutron scattering data. a** X-ray and **b** neutron inelastic scattering measurements along the high symmetry direction Γ-Σ. The faint solid lines are a guide for the eye.

11. Separation of Phonon and Electron Contributions to Thermal Conductivity

To check the compliance or violation of the Wiedemann-Franz law, the phononic and electronic contributions to thermal conductivity need to be separated.

To separate the phononic and electronic contributions, we fit κ_{xx} versus B curves with the following empirical equation:

$$\kappa_{xx}(T,B) = \kappa_{xx}^{ph}(T) + \frac{\kappa_{xx}^{e}(T,B=0\mathrm{T})}{1+\beta_e(T)B^m}. \tag{S17}$$

where $\beta_e(T)$ is proportional to the zero-field electronic mean free path of electrons, and m is related to the nature of the electron scattering[9, 10].

Figures S20b-d shows the gradual suppression of κ_{xx} at high magnetic fields at typical temperatures 100K, 200K and 300K. We can see at 100K, κ_{xx} forms a plateau above 4T, indicating that the electronic thermal conductivity is almost completely suppressed, while at 200K and 300K, the suppression is still in an intermediate state. All the κ_{xx} versus B curves can be fitted well with Eq. (S17) and the fitting process for different temperatures successfully achieves the separation of the phononic and electronic contributions to thermal conductivity. The resulting phononic and electronic thermal conductivities are discussed in detail in the main text. Here we stress that the fitting parameter $\beta_e(T)$ obtained from the fitting shows a typical behavior of thermally elevated electron-phonon scattering, as shown in Figure S20a, and the fitting parameter n fluctuates around 1.35, indicating its constant nature which implies that our fitting process is reasonable.

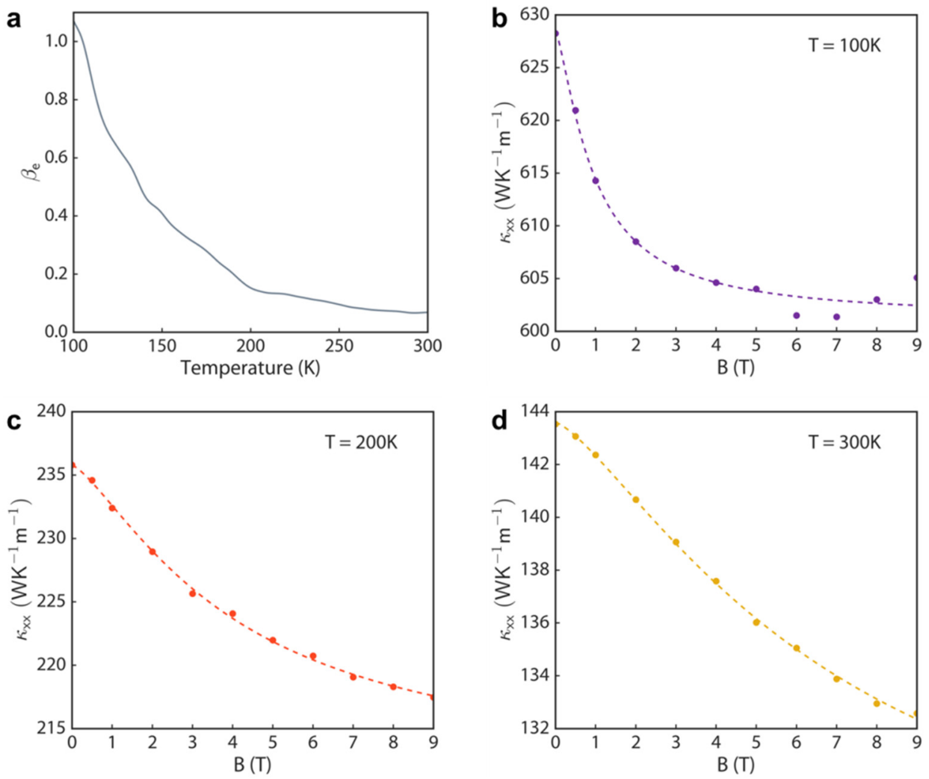

Figure S20. **Data analysis in electronic thermal conductivity. a** The fitting parameter $\beta_e(T)$ as a function of temperature. The fitting for the κ_{xx} versus B curves at **b** 100K, **c** 200K and **d** 300K.

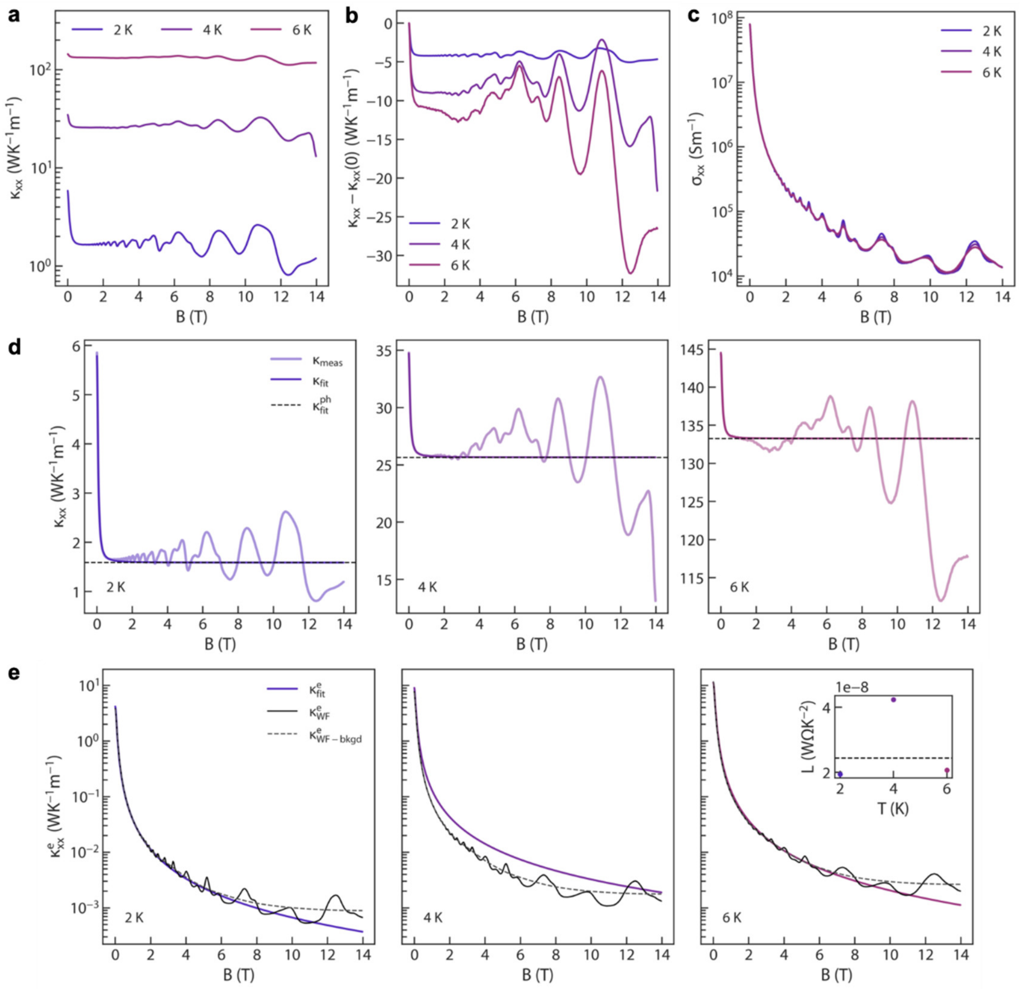

Figure S21. **Wiedemann-Franz law in the low-temperature regime up to *B*=14T. a** Longitudinal thermal conductivity κ_{xx} at 2K, 4K, and 6K as a function of B. **b** Longitudinal thermal conductivity relative to its value at zero field. **c** Longitudinal conductivity σ_{xx} at 2K, 4K, and 6K as a function of B. **d** The result of fitting the empirical equation S17 to κ_{xx} at 2K, 4K, and 6K (left to right). The fitted value of the phonon contribution to κ_{xx} is indicated in each plot by the dashed black line. **e** Comparison of the electronic contribution to κ_{xx} obtained by fitting and by direct calculation using the Wiedemann-Franz law and measured σ_{xx}, showing good agreement at low temperature. Due to the presence of quantum oscillations at high field, a calculation using a smooth background of σ_{xx} is also performed (dashed line).

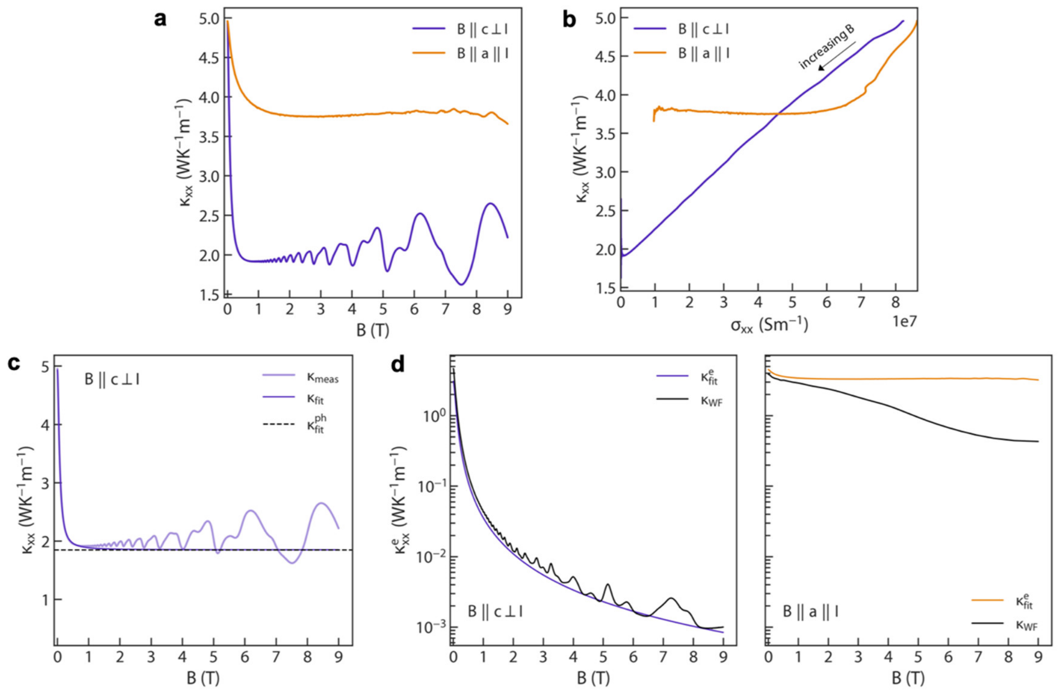

Figure S22. **Comparison between configurations with B || c and B || a. a** Longitudinal thermal conductivity κ_{xx} at the base temperature 2K as a function of B, applied either parallel to the c-axis direction (perpendicular to the current I direction) or parallel to the a-axis direction (parallel to the current direction). **b** κ_{xx} versus the longitudinal conductivity σ_{xx} under the two different configurations. **c** The result of fitting the empirical equation S17 to κ_{xx} in the B || c configuration only. The fitted value of the phonon contribution to κ_{xx} is indicated by the dashed black line. **d** Comparison of the electronic contribution to κ_{xx} obtained by fitting and by direct calculation using the Wiedemann-Franz law and measured σ_{xx} for both configurations. To obtain the fitted value of the electronic contribution in the case of B || a, the phonon contribution obtained by fitting B || c was adjusted by the ratio of sound velocities along the a- and c-axis directions, which were estimated from the calculated phonon dispersion (see Fig. 4c of the main text). The results show good agreement with the Wiedemann-Franz law for the case B || c, but a departure by approximately one order of magnitude at the maximum field for the B || a configuration.

12. Computational Details

All the *ab initio* calculations are performed by Vienna Ab Initio Package (VASP)[11, 12] with projector-augmented-wave (PAW) pseudopotentials and Perdew-Burke-Ernzerhof (PBE) for exchange-correlation energy functional[13]. The geometry optimization of the conventional cell was performed with a $6\times6\times2$ Monkhorst-Pack grid of k-point sampling. The second-order and third-order force constants was calculated using a real space supercell approach with a $3\times3\times1$supercell, same as Ref[14]. The Phonopy package[15] was used to obtain the second-order force constants. The thirdorder.py and ShengBTE packages[16] were used to obtain the third-order force constants and relaxing time approximation was used to calculate the thermal conductivity. A cutoff radius of about 0.42 nm was used, which corresponds to including the fifth nearest neighbor when determining the third-order force constants. To get the equilibrium distribution function and scattering rates using the third-order force constants, the first Brillouin zone was sampled with 30×30×10 mesh.

Supplementary references

1. Skinner B, Fu L. Large, nonsaturating thermopower in a quantizing magnetic field. *Science Advances* 2018, **4**(5)**:** eaat2621.
2. Kozii V, Skinner B, Fu L. Thermoelectric Hall conductivity and figure of merit in Dirac/Weyl materials. *Physical Review B* 2019, **99**(15).
3. Murakawa H, Bahramy MS, Tokunaga M, Kohama Y, Bell C, Kaneko Y*, et al.* Detection of Berry's Phase in a Bulk Rashba Semiconductor. *Science* 2013, **342**(6165)**:** 1490.
4. Sinn H, Alp EE, Alatas A, Barraza J, Bortel G, Burkel E*, et al.* An inelastic X-ray spectrometer with 2.2 meV energy resolution. *Nucl Instrum Meth A* 2001, **467:** 1545-1548.
5. Alatas A, Leu BM, Zhao J, Yavas H, Toellner TS, Alp EE. Improved focusing capability for inelastic X-ray spectrometer at 3-ID of the APS: A combination of toroidal and Kirkpatrick-Baez (KB) mirrors. *Nucl Instrum Meth A* 2011, **649**(1)**:** 166-168.

6. Toellner TS, Alatas A, Said AH. Six-reflection meV-monochromator for synchrotron radiation. *J Synchrotron Radiat* 2011, **18:** 605-611.
7. Sinn H. Spectroscopy with meV energy resolution. *J Phys-Condens Mat* 2001, **13**(34)**:** 7525-7537.
8. Burkel E. Determination of phonon dispersion curves by means of inelastic x-ray scattering. *J Phys-Condens Mat* 2001, **13**(34)**:** 7627-7644.
9. Ocana R, Esquinazi P. Thermal conductivity tensor in YBa2Cu3O7-x: Effects of a planar magnetic field. *Physical Review B* 2002, **66**(6).
10. Uher C. Thermal conductivity of high-Tcsuperconductors. *Journal of Superconductivity* 1990, **3**(4)**:** 337-389.
11. Kresse G, Furthmuller J. Efficiency of ab-initio total energy calculations for metals and semiconductors using a plane-wave basis set. *Comp Mater Sci* 1996, **6**(1)**:** 15-50.
12. Kresse G, Joubert D. From ultrasoft pseudopotentials to the projector augmented-wave method. *Physical Review B* 1999, **59**(3)**:** 1758-1775.
13. Perdew JP, Burke K, Ernzerhof M. Generalized gradient approximation made simple. *Phys Rev Lett* 1996, **77**(18)**:** 3865-3868.
14. Nguyen T, Han F, Andrejevic N, Pablo-Pedro R, Apte A, Tsurimaki Y*, et al.* Topological Singularity Induced Chiral Kohn Anomaly in a Weyl Semimetal. *Phys Rev Lett* 2020, **124**(23)**:** 236401.
15. Togo A, Tanaka I. First principles phonon calculations in materials science. *Scripta Materialia* 2015, **108:** 1-5.
16. Li W, Carrete J, Katcho NA, Mingo N. ShengBTE: A solver of the Boltzmann transport equation for phonons. *Comput Phys Commun* 2014, **185**(6)**:** 1747-1758.
